# Supplementary material for: One-Pot Route from Halogenated Amides to Piperidines and Pyrrolidines
Source: Molecules. 2022 Jul 22;27(15):4698. doi: 10.3390/molecules27154698 (PMC9331508; doi:10.3390/molecules27154698)

One-pot Route from Halogenated Amides to Piperidines and Pyrrolidines

Supplementary Materials

Qiao Song [1, 2, *], Sheng Wang [1], Xiangui Lei [1, 2], Yan Liu [1], Xin Wen [1] and Zhouyu Wang [1, 2, *]

[1] *Department of Chemistry, Xihua University, Chengdu 610039, China*

[2] *Asymmetric Synthesis and Chiral Technology Key Laboratory of Sichuan Province, Yibin 644000, China*

[*] Corresponding author:

E-mail address: songqiao@mail.xhu.edu.cn (Qiao Song)
zhouyuwang77@163.com. (Zhouyu Wang)

Contents:

1. Experimental procedure and characterization data S2

1.1 General procedure B for the synthesis of amide substrates S2

1.2 Characterization S3

2. ^{1}H, ^{13}C NMR and HRMS spectra S10

1. Experimental procedure and characterization data

1.1 General procedure B for the synthesis of amide substrates

Into a dry 25-mL round-bottom flask equipped with a magnetic stirring bar were added successively a primary amine (2.0 mmol, 1.0 equiv.), 20 mL of anhydrous CH_2Cl_2 and triethylamine (4.0 mmol, 2.0 equiv.). After being cooled to 0 °C, acyl halide (2.2 mmol, 1.1 equiv.) was added dropwise. Then the mixture was allowed to warm up to room temperature and stirred for 2h. 1N HCl aqueous solution (10 mL) was added and the organic phase was separated and washed with a saturated aqueous solution of $NaHCO_3$ (2×10 mL). The organic phase was separated and dried over anhydrous Na_2SO_4, filtered and concentrated under reduced pressure. The residue was purified by flash chromatography on silica gel to give the corresponding amide substrates.

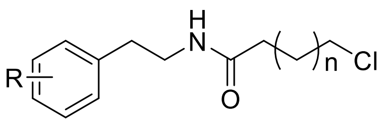

6a,n=2,R=4-Cl
6b,n=2,R=4-F
6c,n=2,R=4-Br
6d,n=2,R=4-CH_3
6e,n=2,R=4-OCH_3
6k,n=1,R=4-Cl
6l,n=1,R=F
6m,n=1,R=Br
6n,n=1,R=OCH_3
6o,n=1,R=CH_3

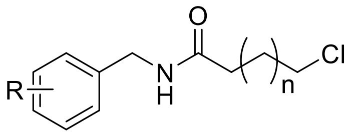

6f,n=1,R=4-NO_2
6g,n=1,R=4-F_3C
6h,n=1,R=4-CH_3
6p,n=1,R=4-NO_2
6q,n=1,R=4-F_3C
6r,n=1,R=H

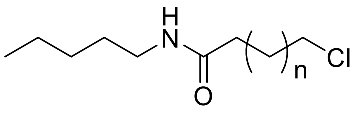

6i,n=2
6s,n=1

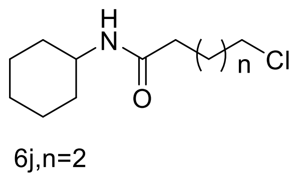

6j,n=2
6t,n=1

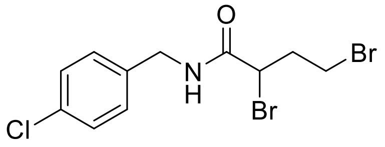

6u

Scheme S1. List of amides used in this work

1.2 Characterization

1-(4-chlorophenethyl) piperidine (**7a**)

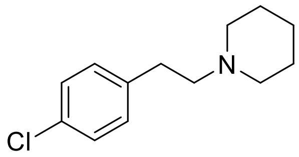

Prepared according to general procedure A; 87% yield; Pale yellow oil; **^{1}H NMR (400 MHz, $CDCl_3$)** δ 7.23 (d, *J* = 8.4 Hz, 2H), 7.12 (d, *J* = 8.4 Hz, 2H), 2.76 (dd, *J* = 9.9, 6.5 Hz, 2H), 2.55 – 2.39 (m, 6H), 1.61 (dt, *J* = 11.1, 5.6 Hz, 4H), 1.49 – 1.39 (m, 2H). **HRMS (ESI)**: calcd for $C_{13}H_{19}ClN^+(M + H)^+$: 224.1201, found: 224.1208. All analytical data were in good accordance with data reported in the literature[32].

1-(4-fluorophenethyl) piperidine (**7b**)

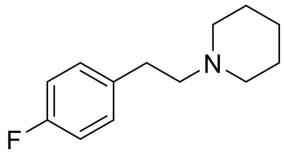

Prepared according to general procedure A; 72% yield; Pale yellow oil; **^{1}H NMR (400 MHz, $CDCl_3$)** δ 7.17 – 7.10 (m, 2H), 7.00 – 6.90 (m, 2H), 2.77 (dd, *J* = 10.1, 6.5 Hz, 2H), 2.56 – 2.36 (m, 6H), 1.65 – 1.56 (m, 4H), 1.51 – 1.41 (m, 2H). **HRMS (ESI)**: calcd for $C_{13}H_{19}FN^+(M + H)^+$: 208.1496, found: 208.1311. All analytical data were in good accordance with data reported in the literature[33].

1-(4-bromophenethyl) piperidine (**7c**)

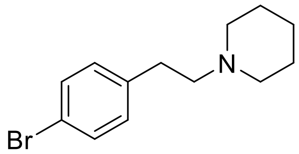

Prepared according to general procedure A; 77% yield; Pale yellow oil; **^{1}H NMR (400 MHz, $CDCl_3$)** δ 7.38 (d, *J* = 8.4 Hz, 2H), 7.07 (d, *J* = 8.4 Hz, 2H), 2.75 (dd, *J* = 9.9, 6.5 Hz, 2H), 2.58 – 2.33 (m, 6H), 1.67 – 1.55 (m, 4H), 1.49 – 1.38 (m, 2H). **HRMS (ESI)**: calcd for $C_{13}H_{19}BrN^+(M + H)^+$: 268.0695, found: 268.0686. All analytical data were in good accordance with data reported in the literature[32].

1-(4-methylphenethyl) piperidine (**7d**)

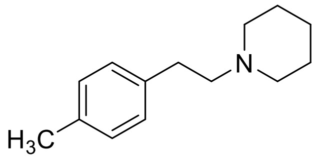

Prepared according to general procedure A; 91% yield; Pale yellow oil; **^{1}H NMR (400 MHz, $CDCl_3$)** δ 7.19 – 7.02 (m, 4H), 3.33 – 2.85 (m, 8H), 2.32 (s, 3H), 2.04 – 1.84 (m, 4H), 1.67 (s, 2H). **HRMS (ESI)**: calcd for $C_{14}H_{22}N^+(M + H)^+$: 204.1747, found: 204.1734. All analytical data were in good accordance with data reported in the literature[34].

1-(4-methoxyphenethyl) piperidine (**7e**)

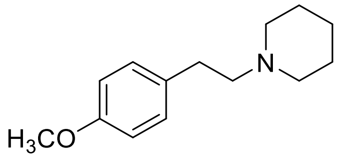

Prepared according to general procedure A; 60% yield; Pale yellow oil; **^{1}H NMR (400 MHz, $CDCl_3$)** δ 7.11 (d, *J* = 8.6 Hz, 2H), 6.82 (d, *J* = 8.6 Hz, 2H), 3.77 (s, 3H), 2.76 (dd, *J* = 10.3, 6.4 Hz, 2H), 2.63 – 2.42 (m, 6H), 1.63 (dt, *J* = 11.2, 5.6 Hz, 4H), 1.52 – 1.40 (m, 2H). **HRMS (ESI)**: calcd for $C_{14}H_{22}NO^+(M + H)^+$: 220.1696, found: 220.1694. All analytical data were in good accordance with data reported in the literature[33].

1-(4-nitrobenzyl) piperidine (**7f**)

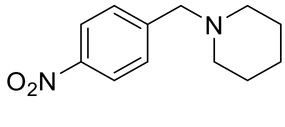

Prepared according to general procedure A; 85% yield; Pale yellow oil; **^{1}H NMR (400 MHz, $CDCl_3$)** δ 8.16 (d, *J* = 8.7 Hz, 2H), 7.50 (d, *J* = 8.7 Hz, 2H), 3.54 (s, 2H), 2.37 (s, 4H), 1.58 (dt, *J* = 11.0, 5.6 Hz, 4H), 1.45 (dd, *J* = 11.2, 5.9 Hz, 2H). **HRMS (ESI)**: calcd for $C_{12}H_{17}N_2O_2^+(M + H)^+$: 221.1285, found: 221.1260. All analytical data were in good accordance with data reported in the literature[35].

1-(4-(trifluoromethyl) benzyl) piperidine (**7g**)

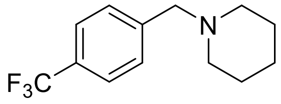

Prepared according to general procedure A; 60% yield; Pale yellow oil; **^{1}H NMR (400 MHz, CDCl₃)** δ 7.56 (d, *J* = 8.1 Hz, 2H), 7.44 (d, *J* = 8.0 Hz, 2H), 3.51 (s, 2H), 2.37 (s, 4H), 1.58 (dt, *J* = 11.0, 5.6 Hz, 4H), 1.45 (dd, *J* = 11.1, 5.8 Hz, 2H). **HRMS (ESI)**: calcd for $C_{13}H_{17}F_3N^+(M + H)^+$: 244.1308, found: 244.1309. All analytical data were in good accordance with data reported in the literature[36].

1-(4-methylbenzyl) piperidine (**7h**)

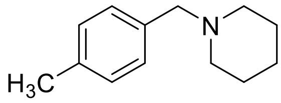

Prepared according to general procedure A; 75% yield; Pale yellow oil; **^{1}H NMR (400 MHz, $CDCl_3$)** δ 7.25 (d, *J* = 7.9 Hz, 2H), 7.13 (d, *J* = 7.8 Hz, 2H), 3.56 (s, 2H), 2.48 (s, 4H), 2.33 (s, 3H), 1.66 (dt, *J* = 11.3, 5.6 Hz, 4H), 1.49 – 1.40 (m, 2H). **HRMS (ESI)**: calcd for $C_{13}H_{20}N^+(M + H)^+$: 190.1590, found: 190.1583. All analytical data were in good accordance with data reported in the literature[37].

1-pentylpiperidine (**7i**)

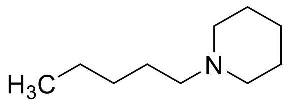

Prepared according to general procedure A; 81% yield; White oil; **^{1}H NMR (400 MHz, $CDCl_3$)** δ 3.81 – 3.16 (m, 2H), 3.11 – 2.58 (m, 4H), 1.94 (d, *J* = 4.4 Hz, 4H), 1.85 – 1.70 (m, 3H), 1.53 – 1.20 (m, 5H), 0.91 (t, *J* = 6.8 Hz, 3H). **HRMS (ESI)**: calcd for $C_{10}H_{22}N^+(M + H)^+$: 156.1747, found: 156.1748. All analytical data were in good accordance with data reported in the literature[38].

1-cyclohexylpiperidine (**7j**)

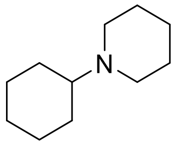

Prepared according to general procedure A; 83% yield; Orange oil; **^{1}H NMR (400**

MHz, $CDCl_3$) δ 2.48 (s, 4H), 2.27 – 2.15 (m, 1H), 1.79 (d, *J* = 34.5 Hz, 4H), 1.55 (s, 5H), 1.39 (s, 2H), 1.27 – 1.13 (m, 4H), 1.12 – 0.95 (m, 1H). **HRMS (ESI)**: calcd for $C_{11}H_{22}N^+(M + H)^+$: 168.1747, found: 168.1740. All analytical data were in good accordance with data reported in the literature[39].

1-(4-chlorophenethyl) pyrrolidine (**7k**)

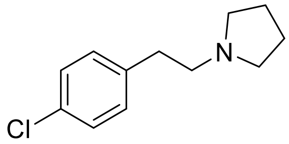

Prepared according to general procedure A; 80% yield; Pale yellow oil; **^{1}H NMR (400 MHz, $CDCl_3$)** δ 7.38 (d, *J* = 8.3 Hz, 2H), 7.07 (d, *J*= 8.2 Hz, 2H), 2.79 (dd, *J*= 10.1, 5.6 Hz, 2H), 2.74 – 2.67 (m, 2H), 2.62 (m, 4H), 1.91 – 1.78 (m, 4H). **HRMS (ESI)**: calcd for $C_{12}H_{17}ClN^+(M + H)^+$: 210.1044, found: 210.1038. All analytical data were in good accordance with data reported in the literature[40].

1-(4-fluorophenethyl) pyrrolidine (**7l**)

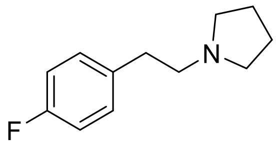

Prepared according to general procedure A; 75% yield; Pale yellow oil; **^{1}H NMR (400 MHz, $CDCl_3$)** δ 7.18 – 7.11(m, 2H), 7.01 – 6.90 (m, 2H), 2.88 – 2.79 (m, 2H), 2.79 – 2.71 (m, 2H), 2.70 – 2.62 (m, 4H), 1.90 – 1.76 (m, 4H). **HRMS (ESI)**: calcd for $C_{12}H_{17}FN^+(M + H)^+$: 194.1340, found: 194.1333. All analytical data were in good accordance with data reported in the literature[41].

1-(4-bromophenethyl) pyrrolidine (**7m**)

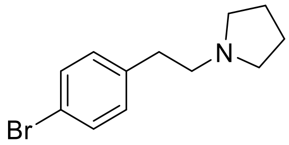

Prepared according to general procedure A; 70% yield; Pale yellow oil; **^{1}H NMR (400 MHz, $CDCl_3$)** δ 7.22 (d, *J* = 8.4 Hz, 2H), 7.12 (d, *J* = 8.3 Hz, 2H), 2.85 – 2.79 (m, 2H), 2.78 – 2.72 (m, 2H), 2.66 (t, *J* = 5.4 Hz, 4H), 1.90 – 1.77 (m, 4H). **HRMS (ESI)**: calcd for $C_{12}H_{17}BrN^+(M + H)^+$: 254.0539, found: 254.0526. All analytical data were in

good accordance with data reported in the literature[42].

1-(4-methoxyphenethyl) pyrrolidine (**7n**)

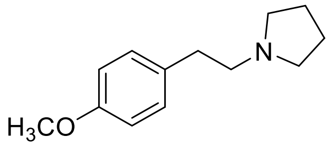

Prepared according to general procedure A; 78% yield; Pale yellow oil; **^{1}H NMR (400 MHz, $CDCl_3$)** δ 7.15 (d, *J* = 8.6 Hz, 2H), 6.82 (d, *J* = 8.6 Hz, 2H), 3.76 (s, 3H), 3.46 – 3.24 (m, 6H), 3.08 – 2.91 (m, 2H), 2.21 – 1.91 (m, 4H). **HRMS (ESI)**: calcd for $C_{13}H_{20}NO^+(M + H)^+$: 206.1539, found: 206.1535. All analytical data were in good accordance with data reported in the literature[43].

1-(4-methylphenethyl) pyrrolidine (**7o**)

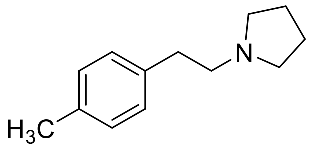

Prepared according to general procedure A; 86% yield; Pale yellow oil; **^{1}H NMR (400 MHz, $CDCl_3$)** δ 7.13 (s, 4H), 3.49 – 3.24 (m, 6H), 3.17 – 2.92 (m, 2H), 2.32 (s, 3H), 2.20 – 2.06 (m, 4H). **HRMS (ESI)**: calcd for $C_{13}H_{20}N^+(M + H)^+$: 190.1590, found: 190.1589. All analytical data were in good accordance with data reported in the literature[42].

1-(4-nitrobenzyl) pyrrolidine (**7p**)

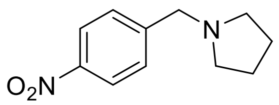

Prepared according to general procedure A; 80% yield; Pale yellow oil; **^{1}H NMR (400 MHz, $CDCl_3$)** δ 8.16 (d, *J* = 8.8 Hz, 2H), 7.50 (d, *J* = 8.8 Hz, 2H), 3.70 (s, 2H), 2.59 – 2.45 (m, 4H), 1.83 – 1.78 (m, 4H). **HRMS (ESI)**: calcd for $C_{11}H_{15}N_2O_2^+(M + H)^+$: 207.1128, found: 207.1114. All analytical data were in good accordance with data reported in the literature[44].

1-(4-(trifluoromethyl) benzyl) pyrrolidine (**7q**)

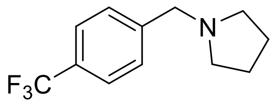

Prepared according to general procedure A; 84% yield; Pale yellow oil; **^{1}H NMR (400 MHz, $CDCl_3$)** δ 7.56 (d, *J* = 8.1 Hz, 2H), 7.45 (d, *J* = 8.0 Hz, 2H), 3.67 (s, 2H), 2.57 – 2.46 (m, 4H), 1.86 – 1.74 (m, 4H). **HRMS (ESI)**: calcd for $C_{12}H_{15}F_3N^+(M + H)^+$: 230.1151, found: 230.1128. All analytical data were in good accordance with data reported in the literature[45].

1-benzylpyrrolidine (**7r**)

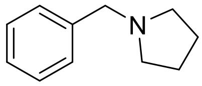

Prepared according to general procedure A; 65% yield; Pale yellow oil; **^{1}H NMR (400 MHz, $CDCl_3$)** δ 7.38 – 7.28 (m, 4H), 7.27 – 7.21 (m, 1H), 3.62 (s, 2H), 2.57 – 2.46 (m, 4H), 1.87 – 1.72 (m, 4H). **HRMS (ESI)**: calcd for $C_{11}H_{16}N^+(M + H)^+$: 162.1277, found: 162.1273. All analytical data were in good accordance with data reported in the literature[46].

1-pentylpyrrolidine (**7s**)

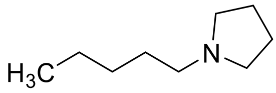

Prepared according to general procedure A; 76% yield; Pale yellow oil; **^{1}H NMR (400 MHz, $CDCl_3$)** δ 3.79 (dd, *J* = 10.0, 4.8 Hz, 2H), 3.19 – 3.04 (m, 2H), 2.96 (d, *J*= 7.3 Hz, 2H), 2.27 – 2.08 (m, 4H), 1.86 – 1.74 (m, 2H), 1.42 – 1.32 (m, 4H), 1.01 – 0.87 (m, 3H). **HRMS (ESI)**: calcd for $C_9H_{20}N^+(M + H)^+$: 142.1590, found: 142.1573. All analytical data were in good accordance with data reported in the literature[47].

1-cyclohexylpyrrolidine (**7t**)

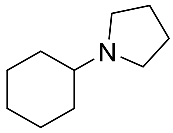

Prepared according to general procedure A; 80% yield; Pale yellow oil; **^{1}H NMR**

(400 MHz, $CDCl_3$) δ 3.34 (s, 3H), 2.97 – 2.87 (m, 1H), 2.11 (d, *J* = 14.8 Hz, 6H), 1.91 (d, *J* = 13.2 Hz, 2H), 1.73 – 1.50 (m, 3H), 1.47 – 1.07 (m, 4H). **HRMS (ESI)**: calcd for $C_{10}H_{20}N^+(M + H)^+$: 154.1590, found: 154.1581. All analytical data were in good accordance with data reported in the literature[48].

3-bromo-1-(4-chlorobenzyl) pyrrolidine (**7u**)

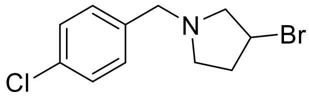

Prepared according to general procedure A; 83% yield; Pale yellow solid; **^{1}H NMR (400 MHz, $CDCl_3$)** δ 7.50 (d, *J* = 8.5 Hz, 2H), 7.40 (d, *J* = 8.5 Hz, 2H), 4.63 (m, 1H), 4.42 – 4.30 (q, 2H), 4.04 (dd, *J* = 13.2, 5.9 Hz, 1H), 3.79 – 3.57 (m, 1H), 3.49 – 3.33 (m,2H), 2.83 – 2.62 (m, 1H), 2.42 (m, 1H). **^{13}C NMR (101 MHz, $CDCl_3$)** δ 136.5, 131.8, 129.8, 128.8, 61.8, 59.9, 53.1, 43.0, 35.8. **HRMS (ESI)**: calcd for $C_{11}H_{14}BrClN^+(M + H)^+$: 273.9993, found: 273.9989.

2. ^{1}H, ^{13}C NMR and HRMS spectra

^{1}H NMR spectrum of 7a

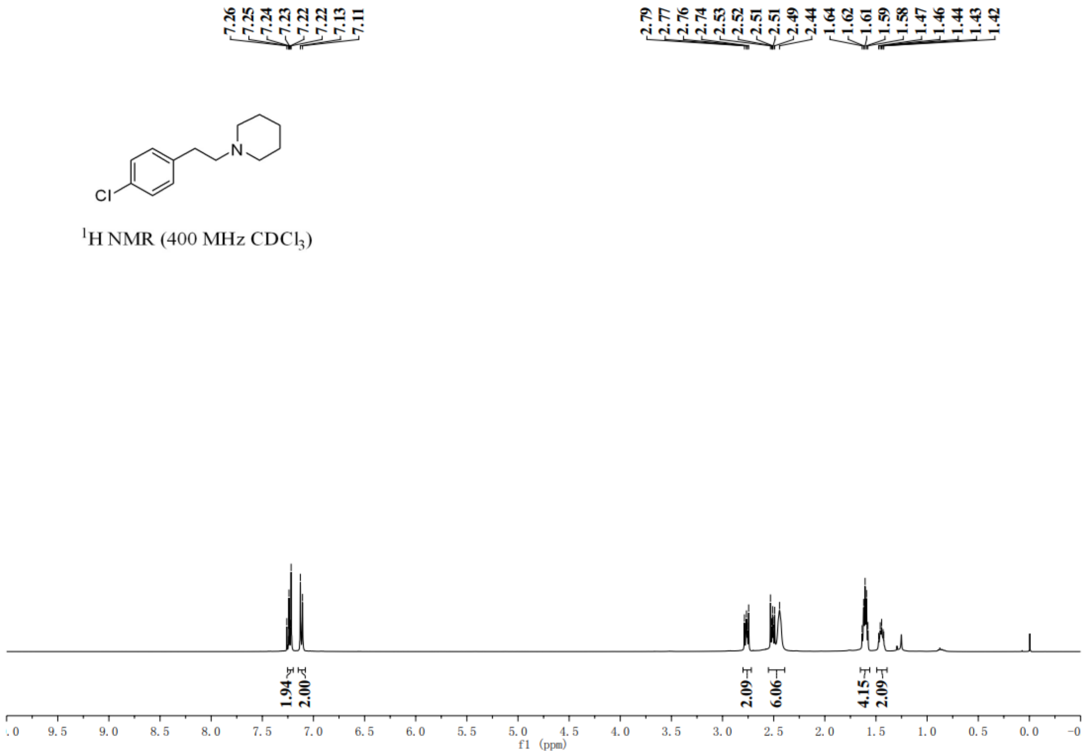

HRMS spectrum of 7a

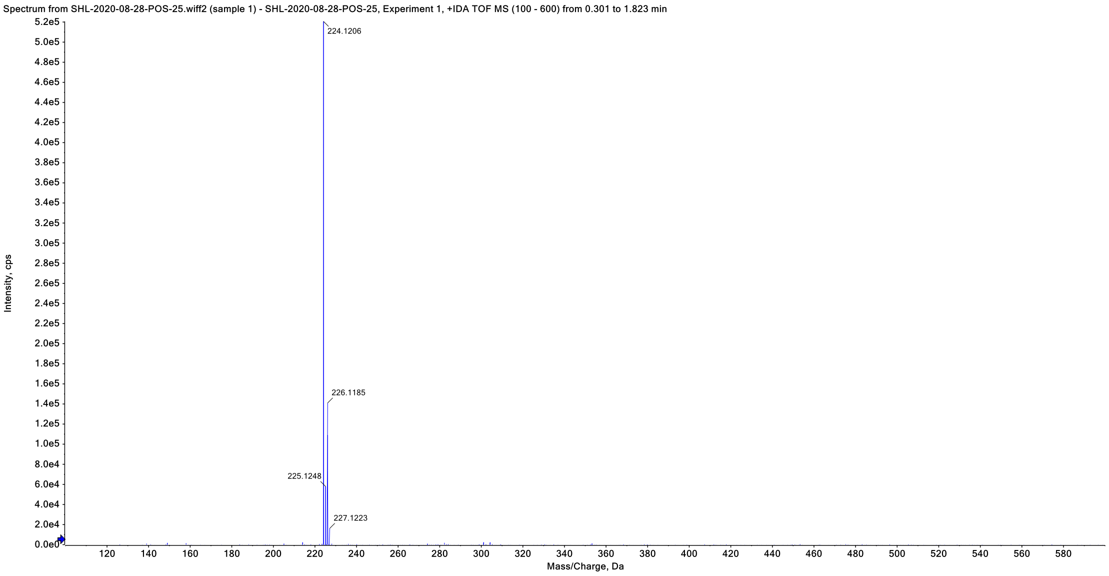

^{1}H NMR spectrum of 7b

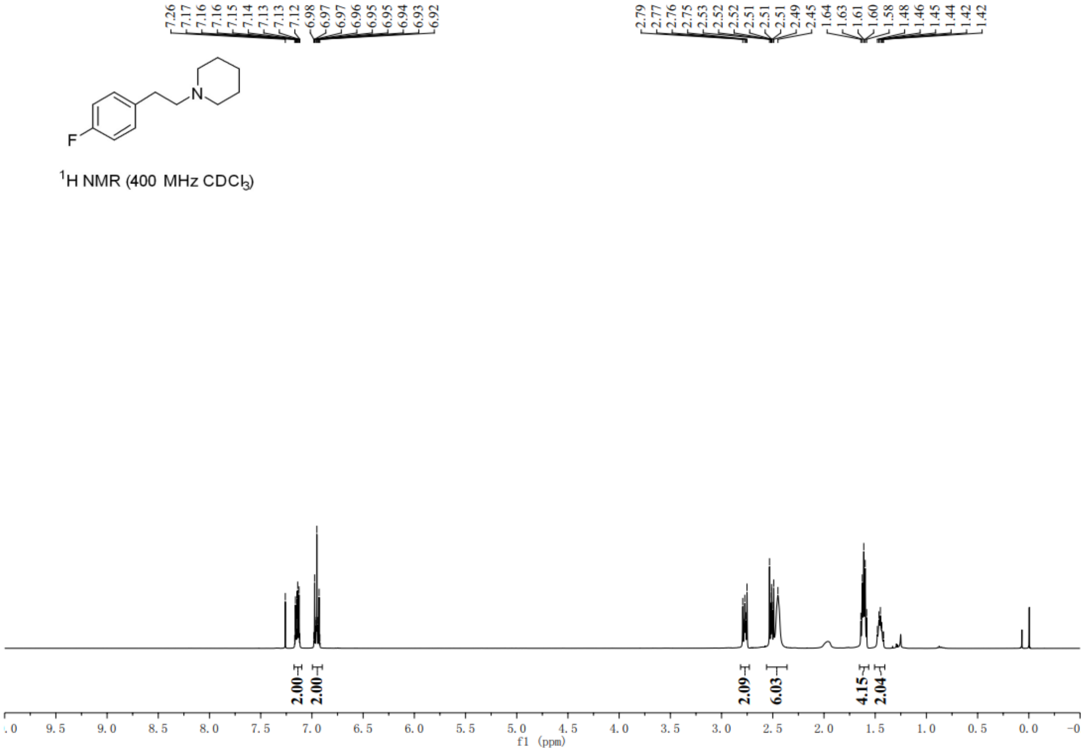

HRMS spectrum of 7b

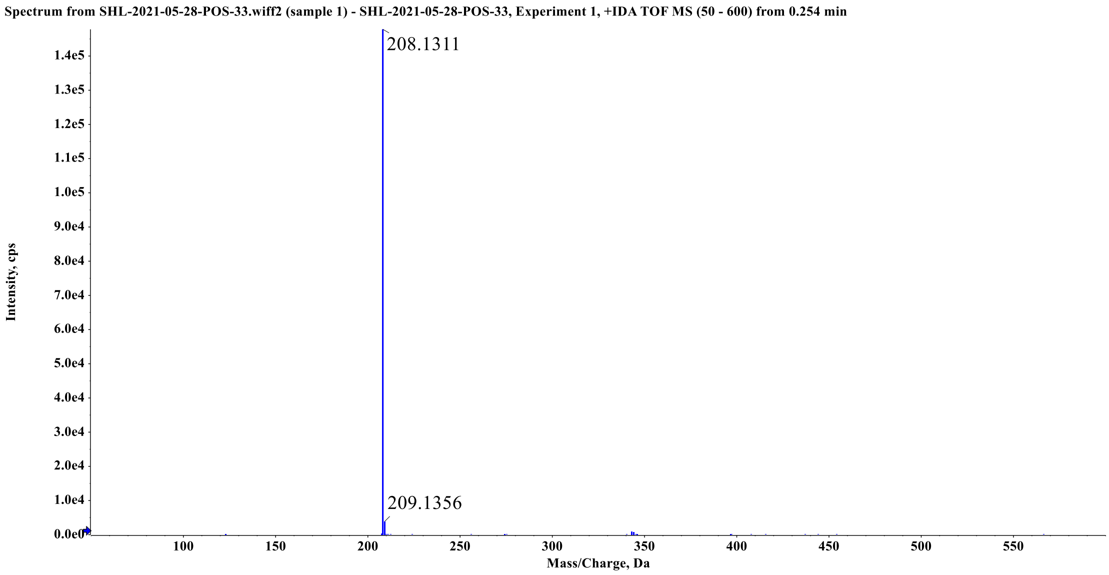

^{1}H NMR spectrum of 7c

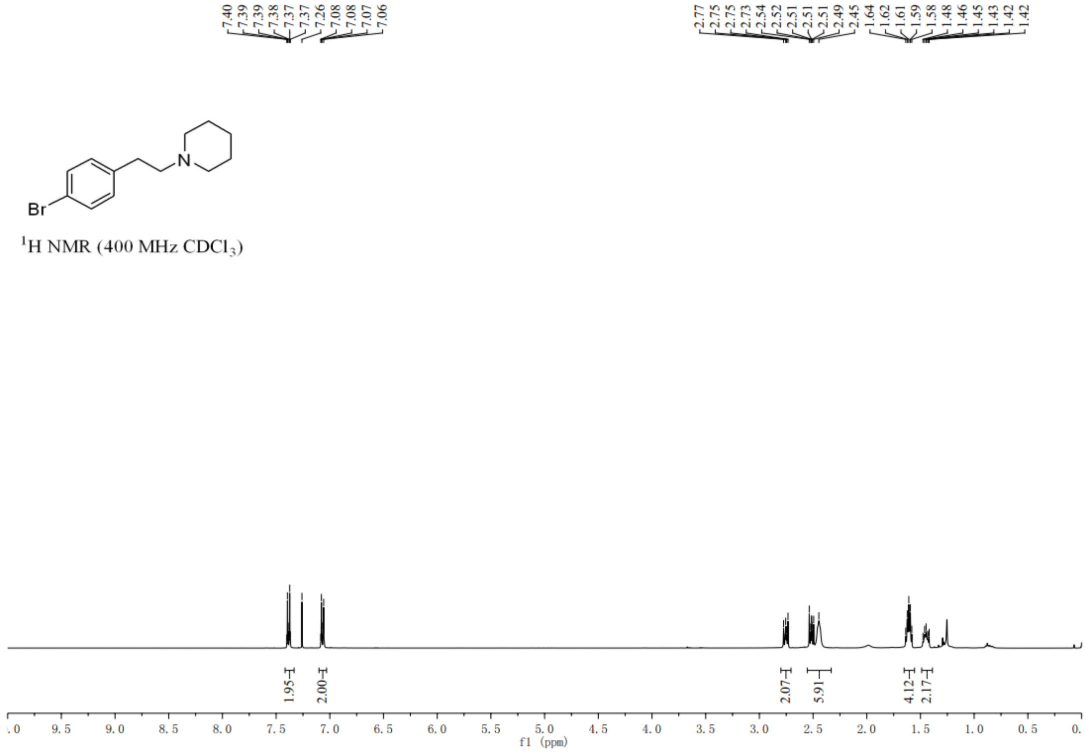

HRMS spectrum of 7c

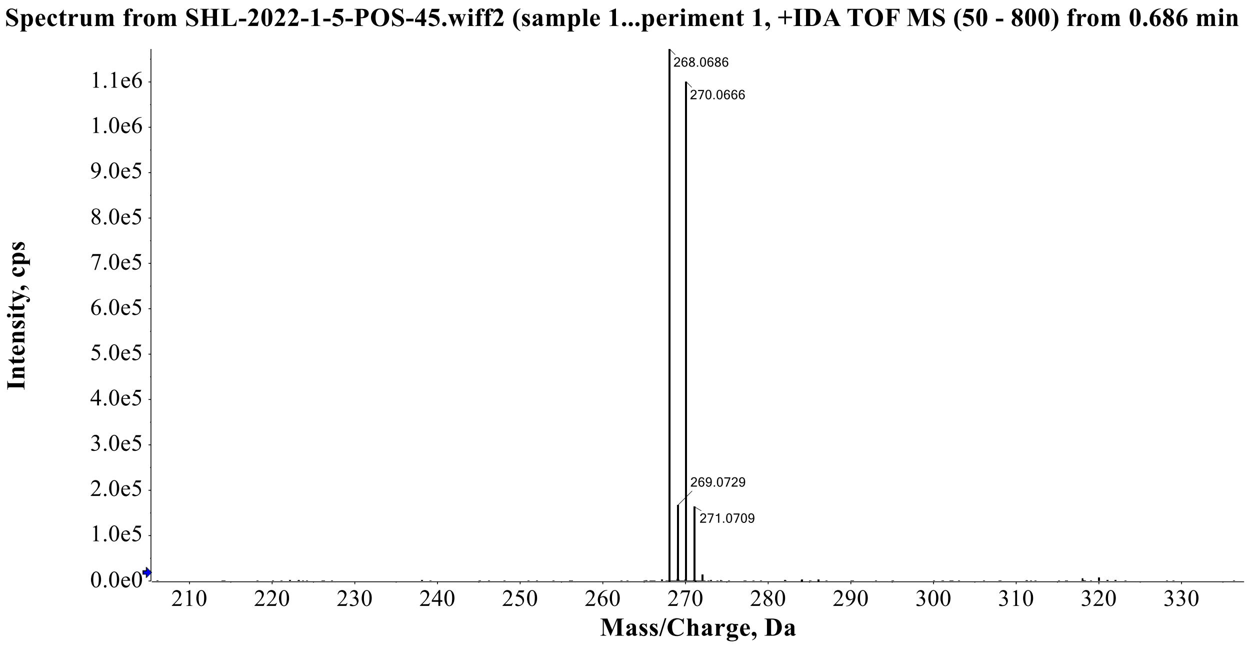

^{1}H NMR spectrum of 7d

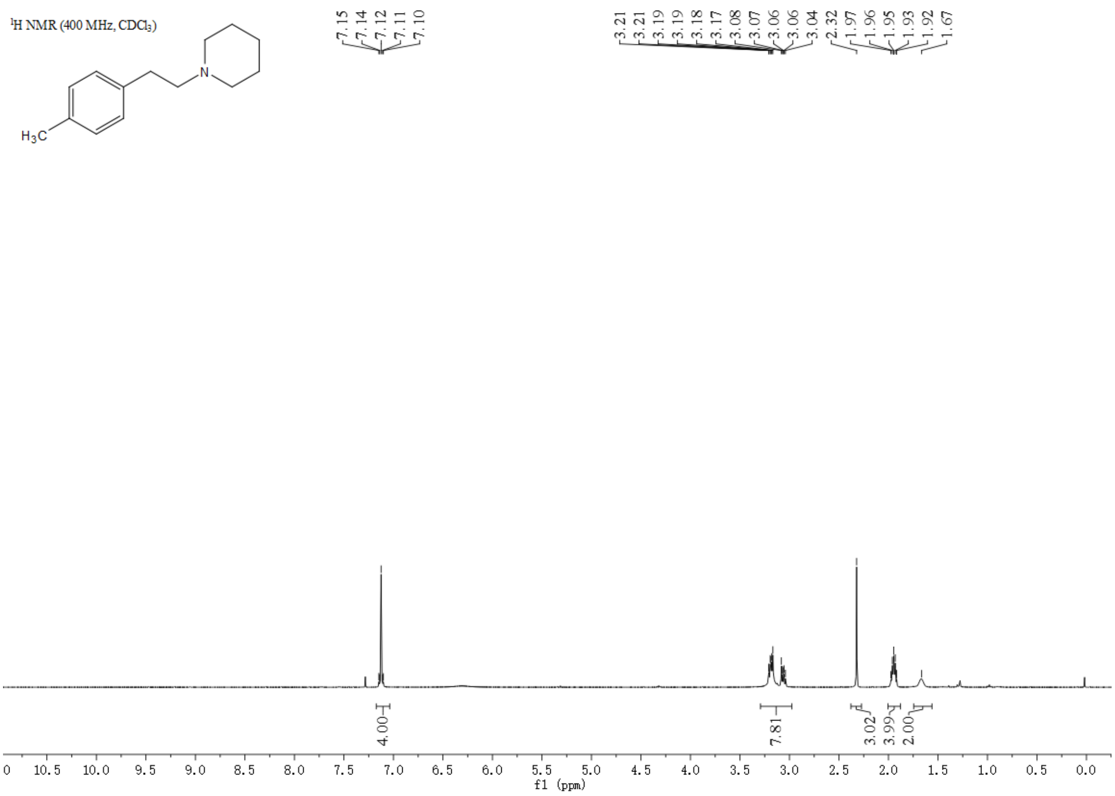

HRMS spectrum of 7d

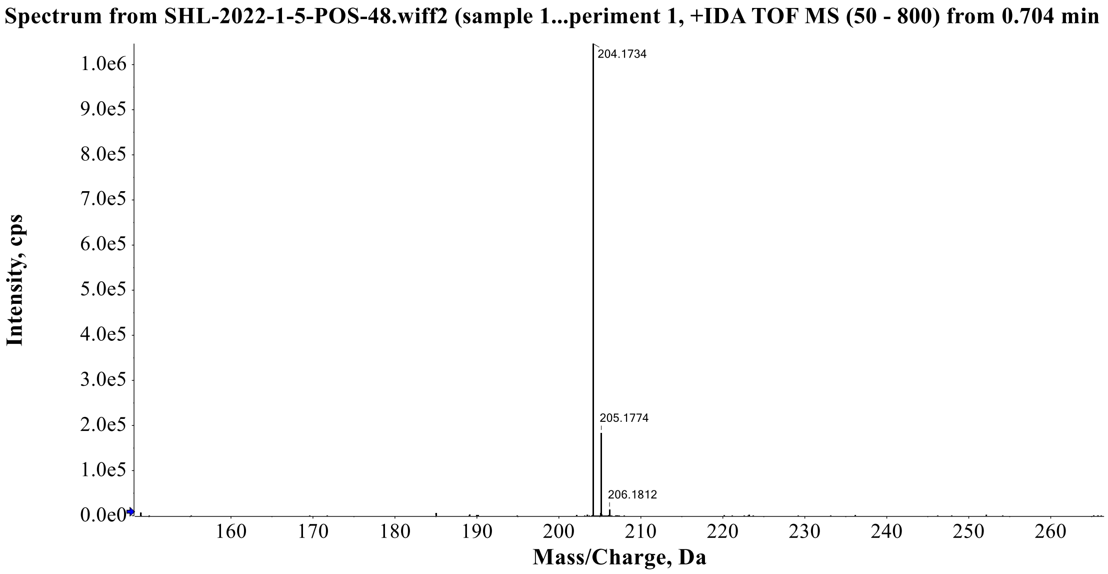

^{1}H NMR spectrum of 7e

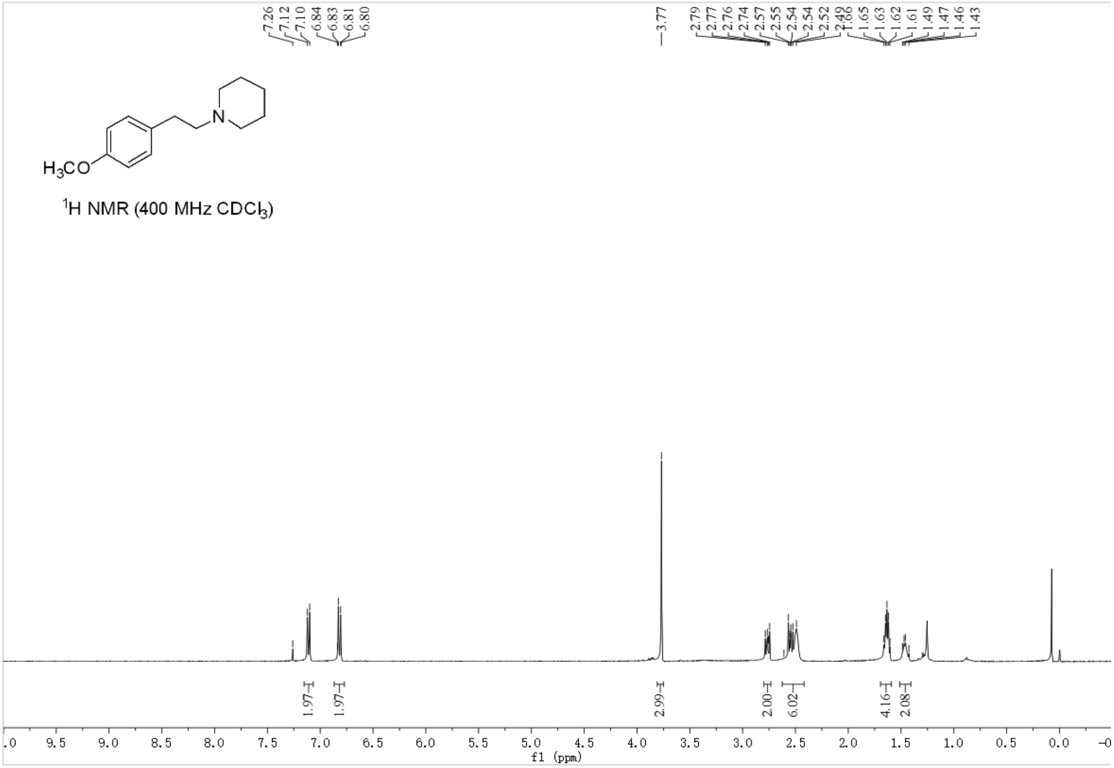

HRMS spectrum of 7e

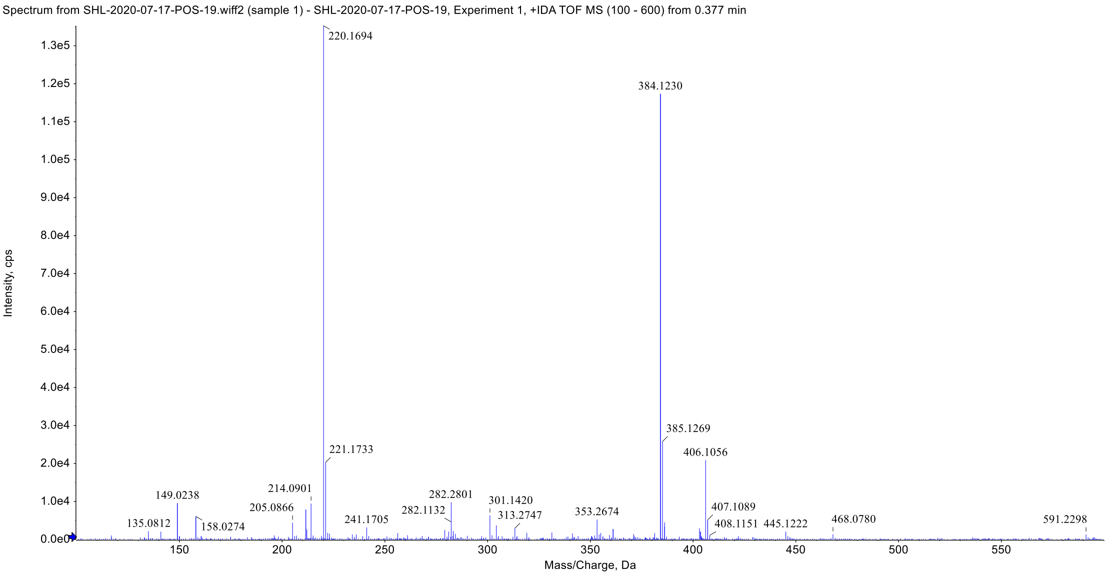

^{1}H NMR spectrum of 7f

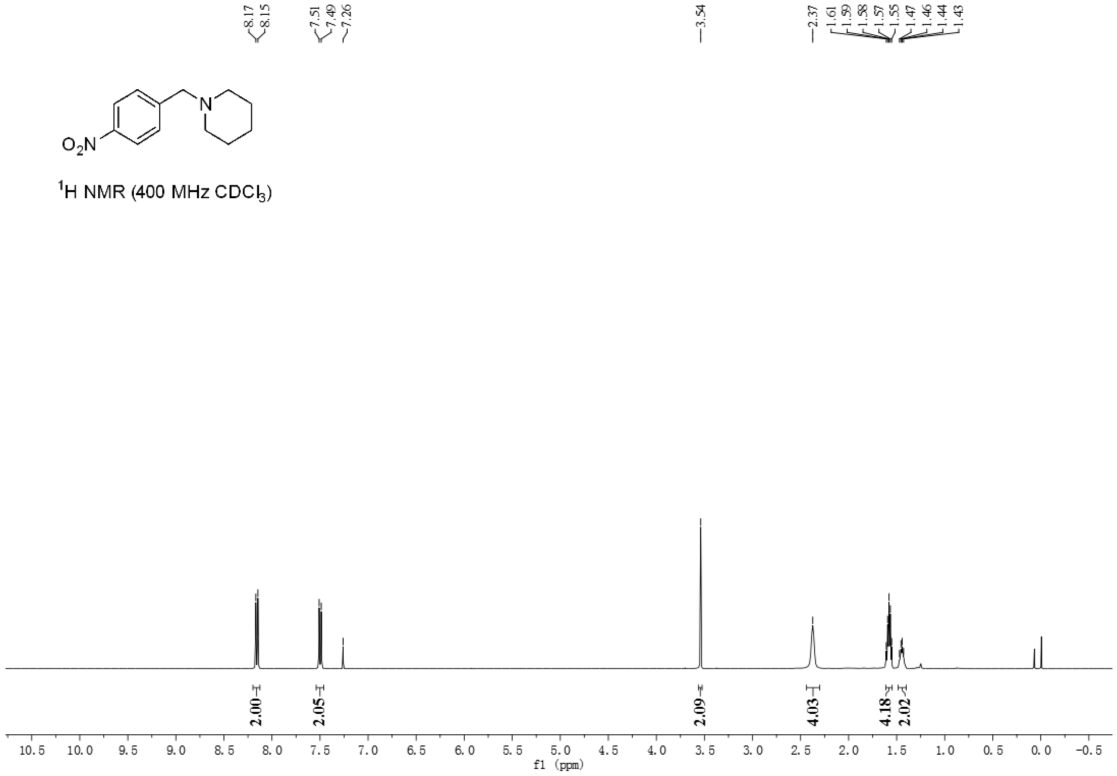

HRMS spectrum of 7f

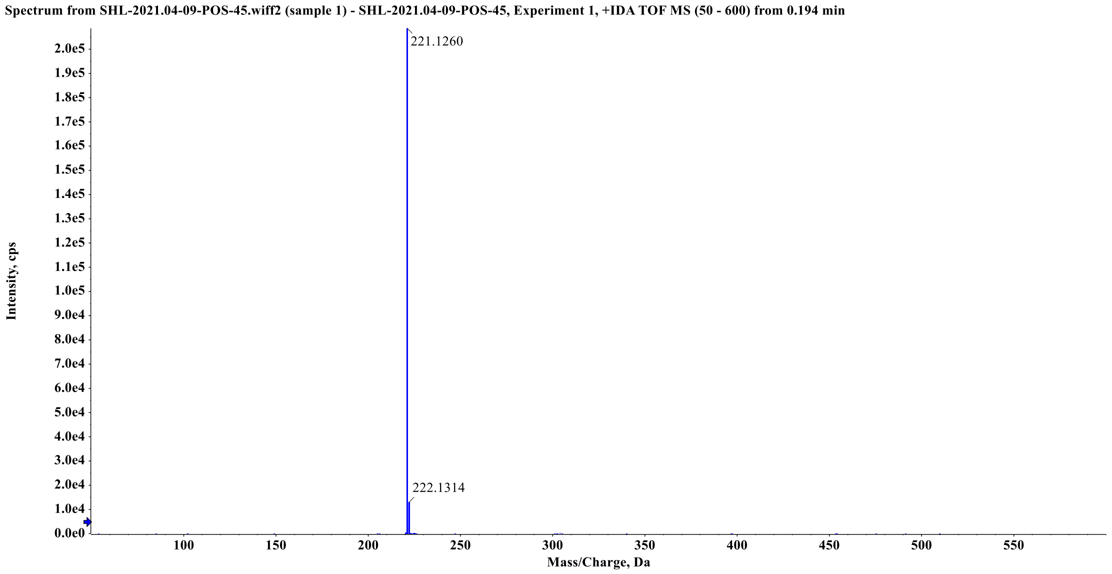

^{1}H NMR spectrum of 7g

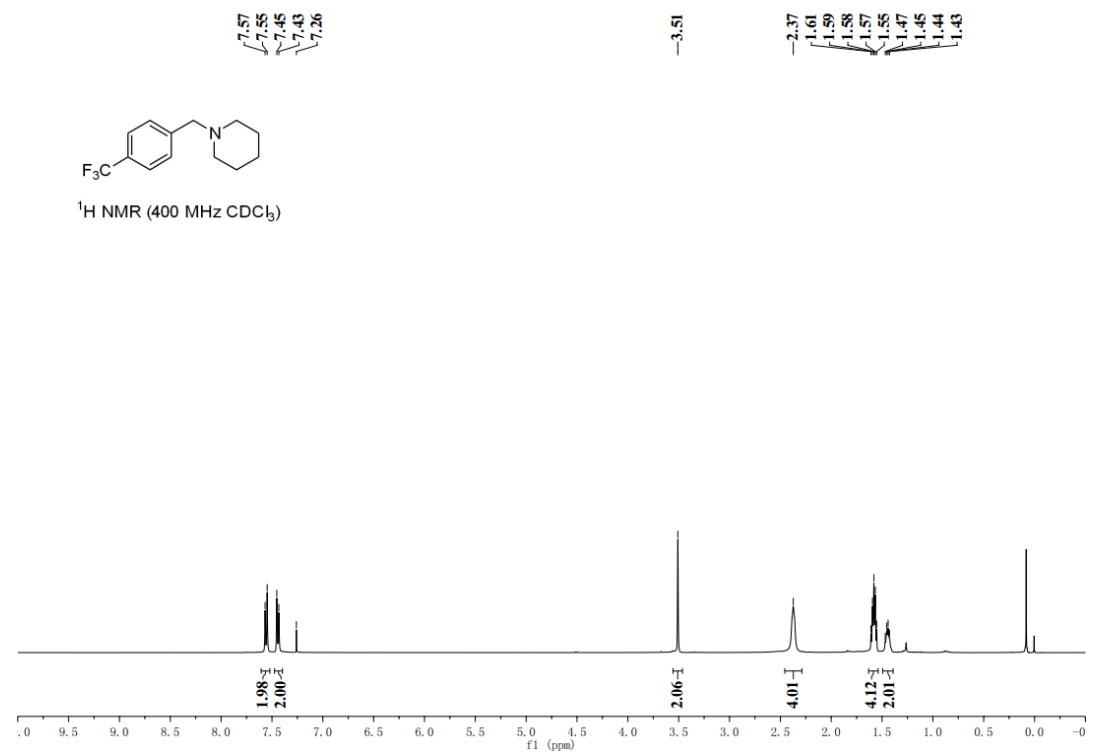

HRMS spectrum of 7g

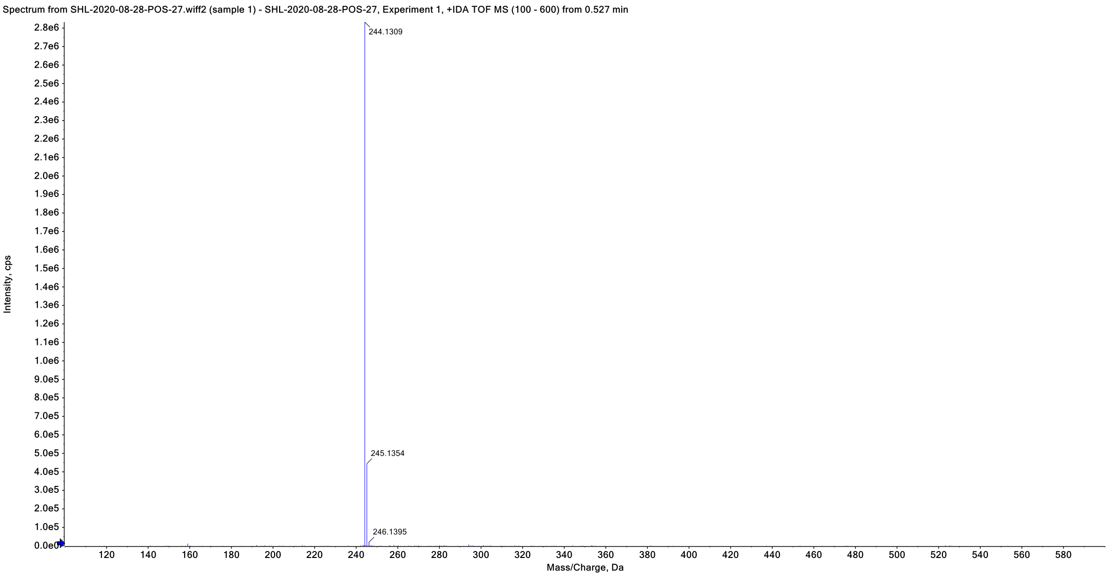

^{1}H NMR spectrum of 7h

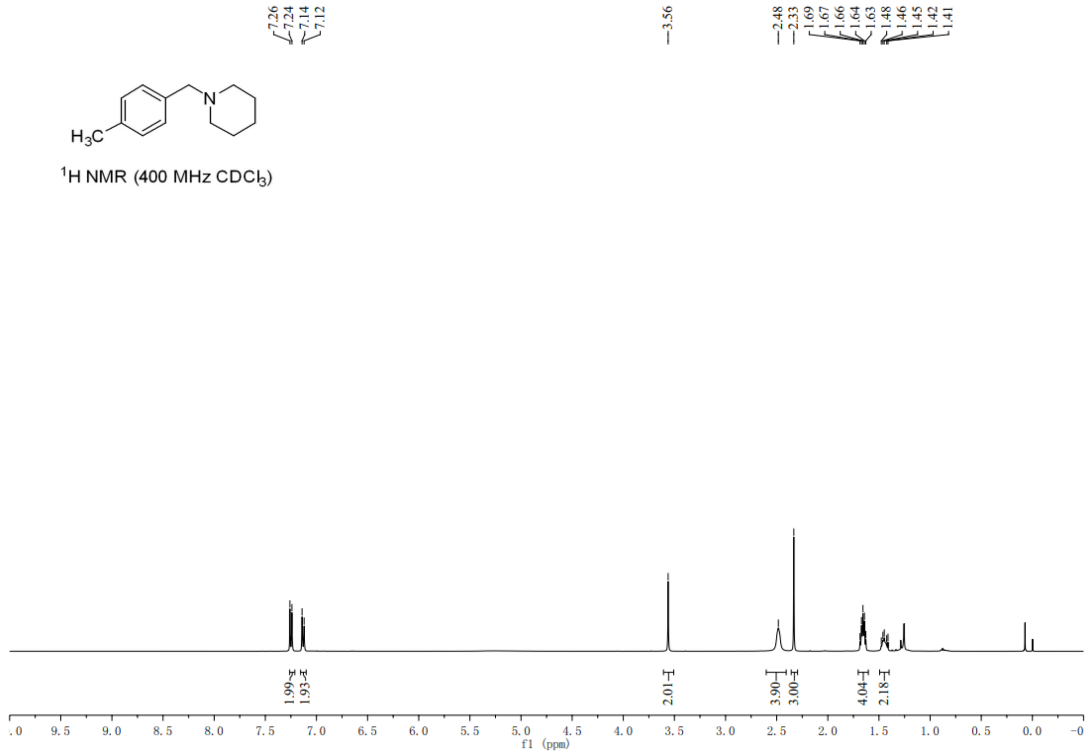

HRMS spectrum of 7h

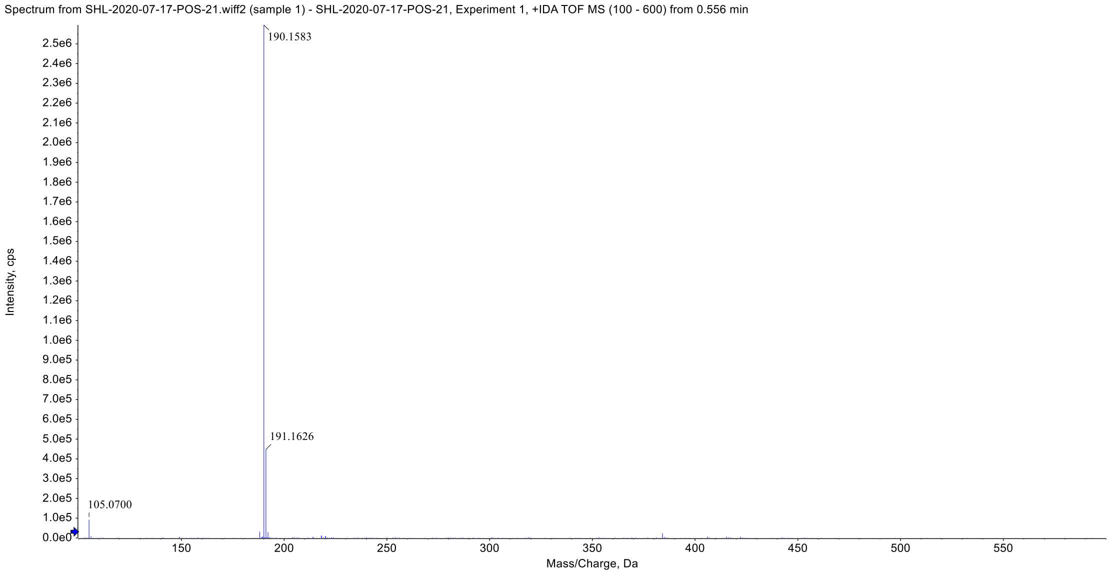

^{1}H NMR spectrum of 7i

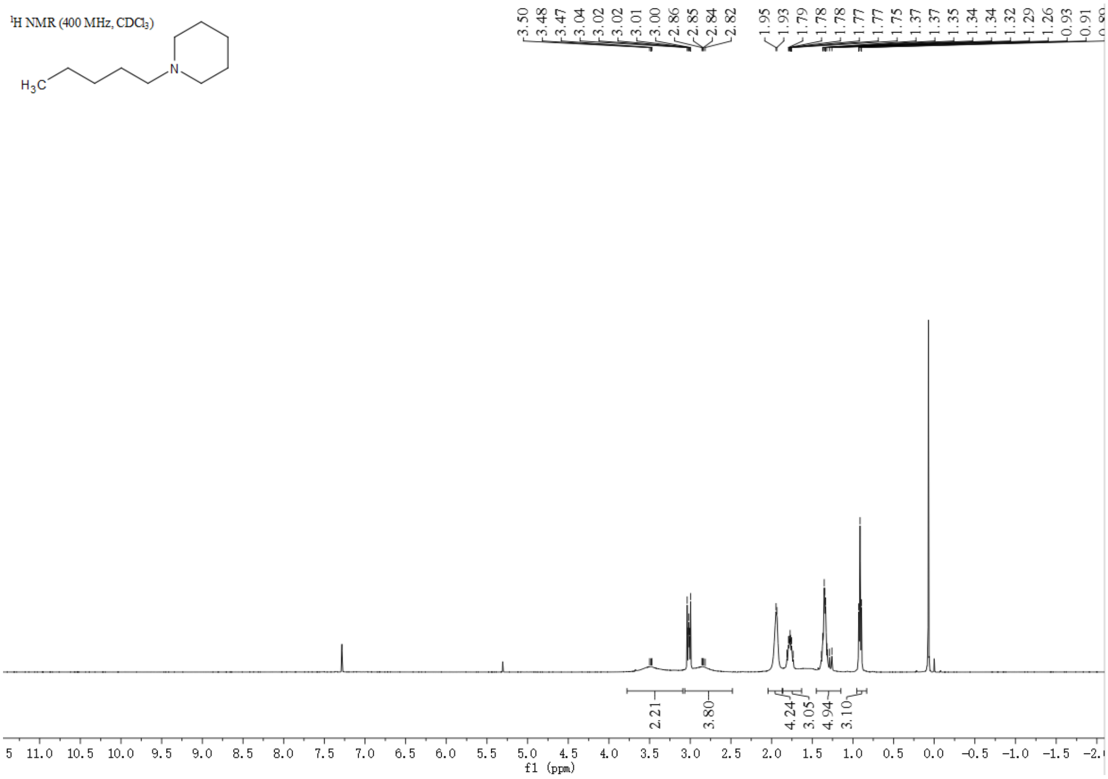

HRMS spectrum of 7i

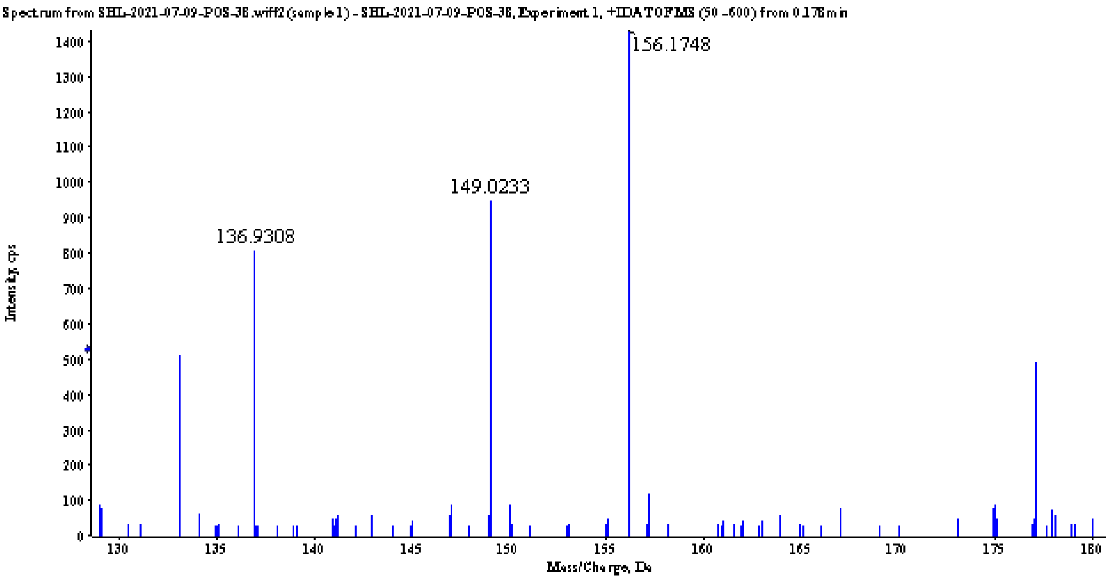

^{1}H NMR spectrum of 7j

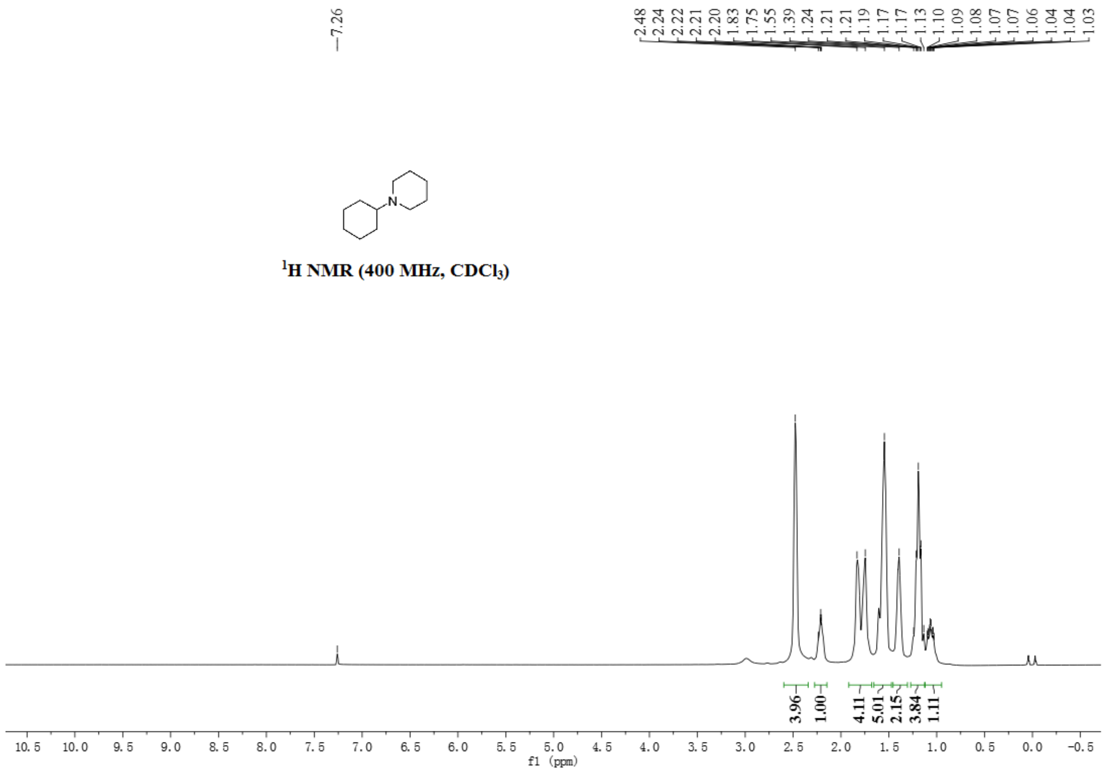

HRMS spectrum of 7j

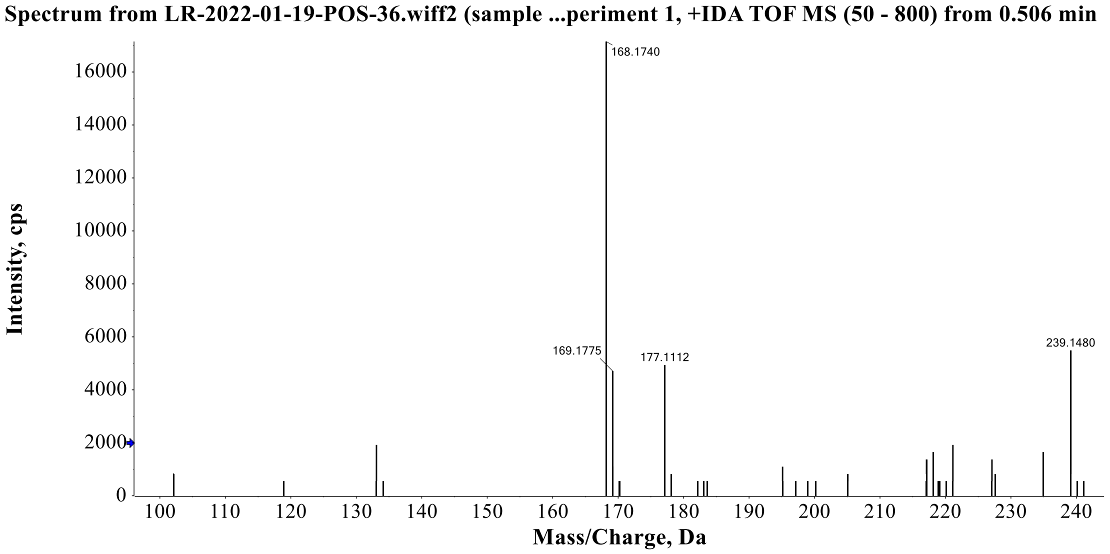

^{1}H NMR spectrum of 7k

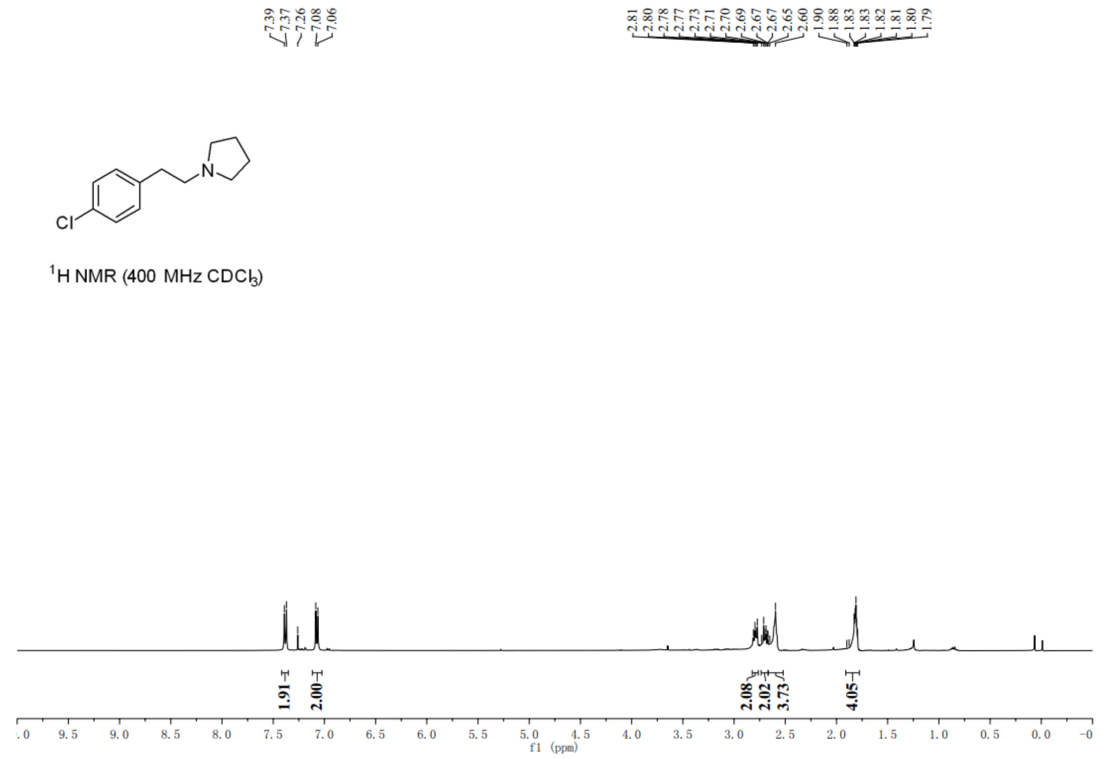

HRMS spectrum of 7k

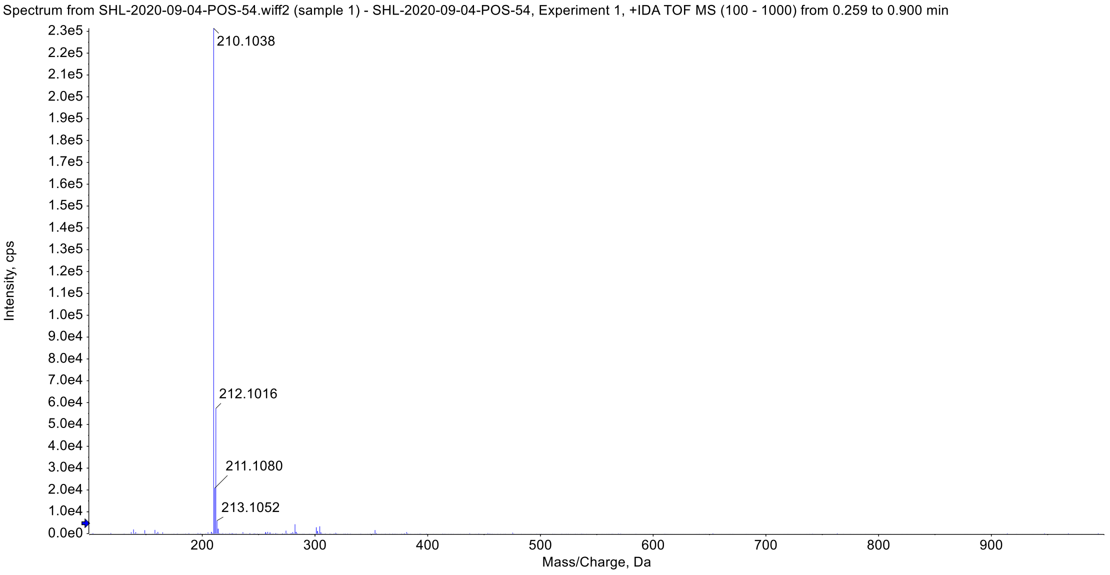

^{1}H NMR spectrum of 7l

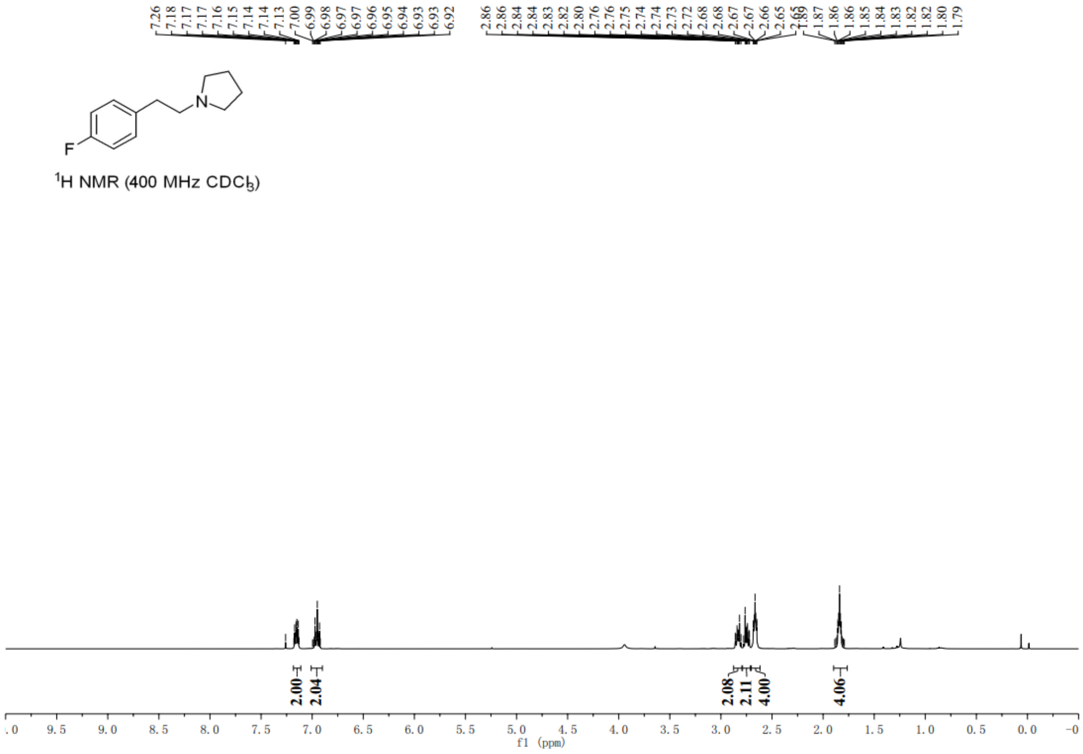

HRMS spectrum of 7l

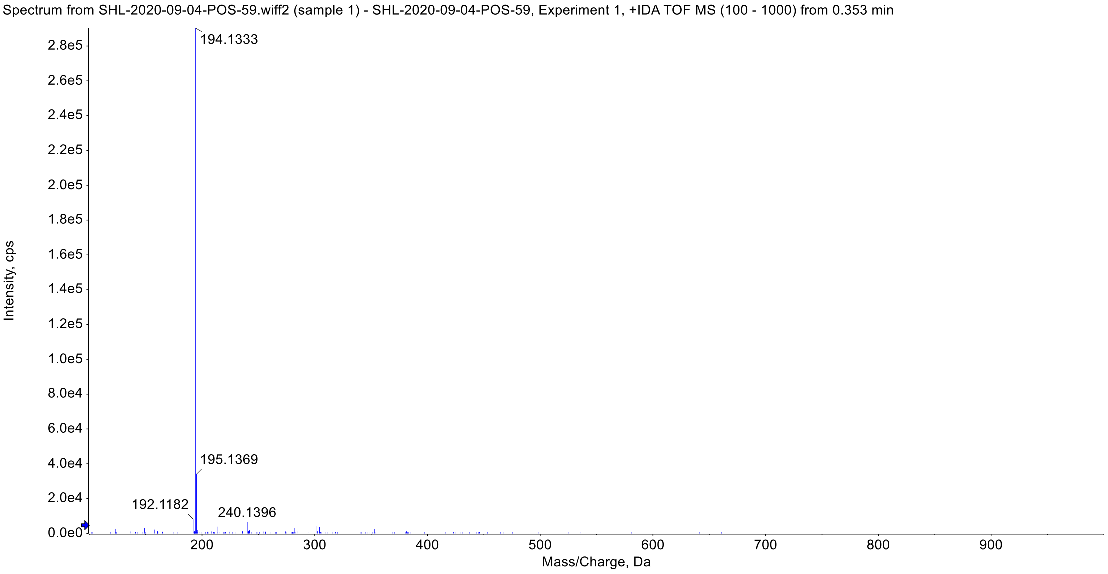

^{1}H NMR spectrum of 7m

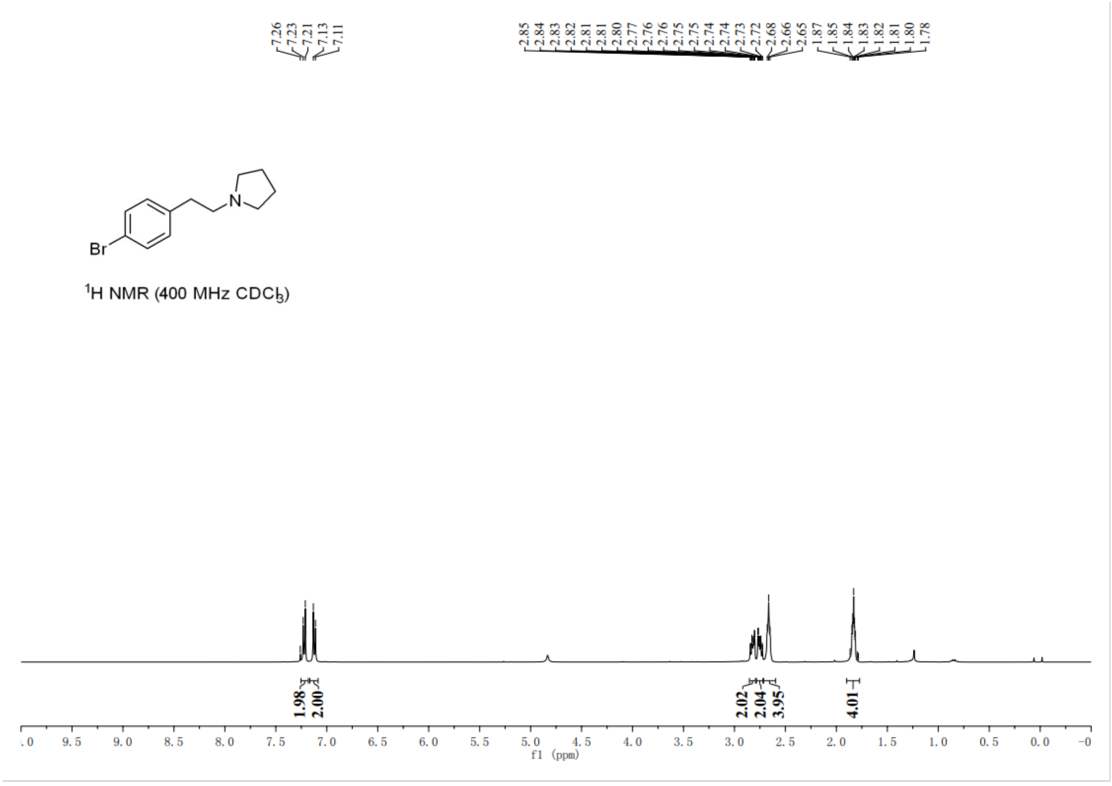

HRMS spectrum of 7m

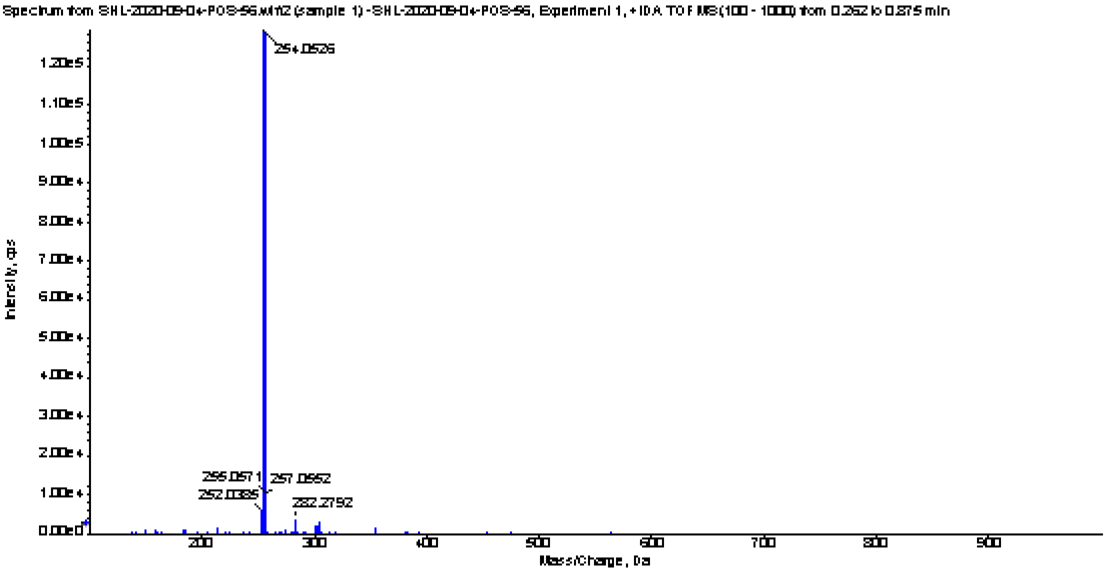

^{1}H NMR spectrum of 7n

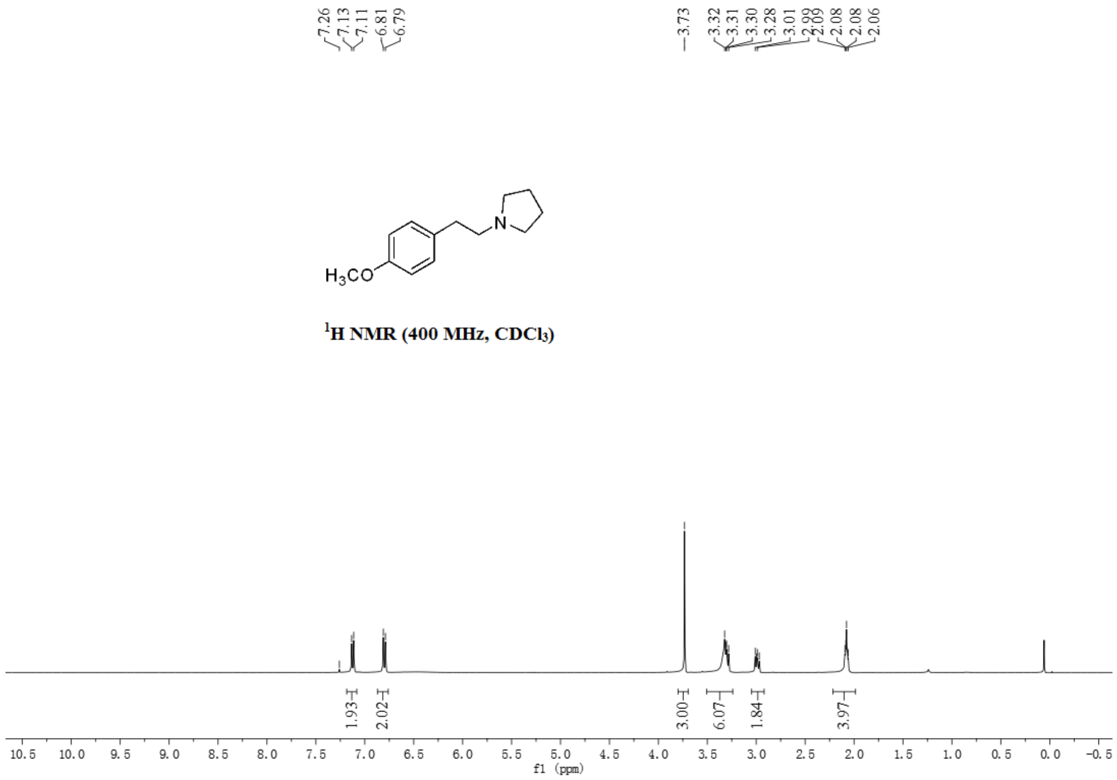

HRMS spectrum of 7n

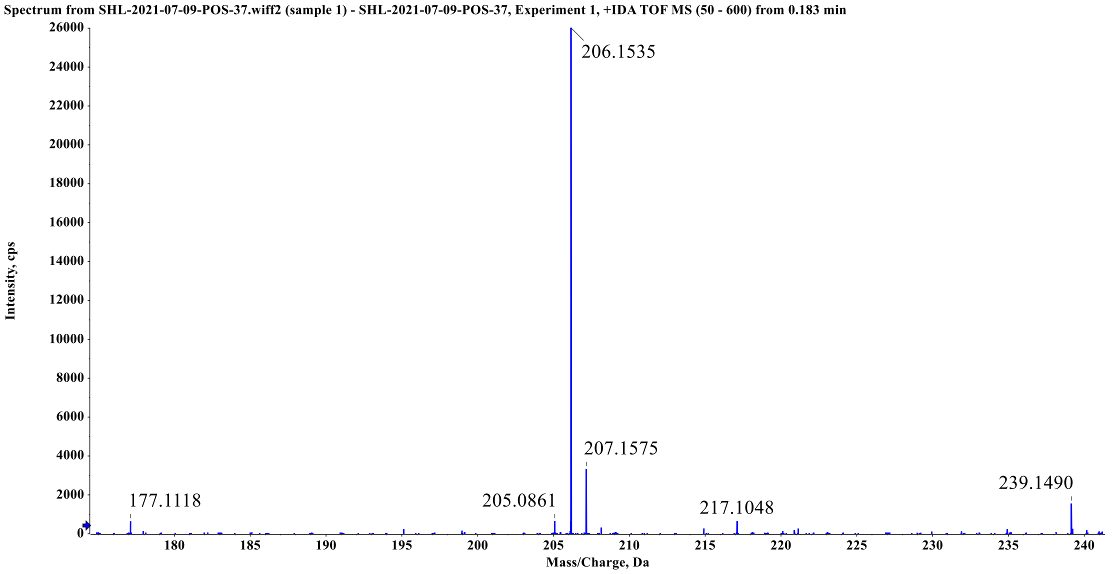

^{1}H NMR spectrum of 7o

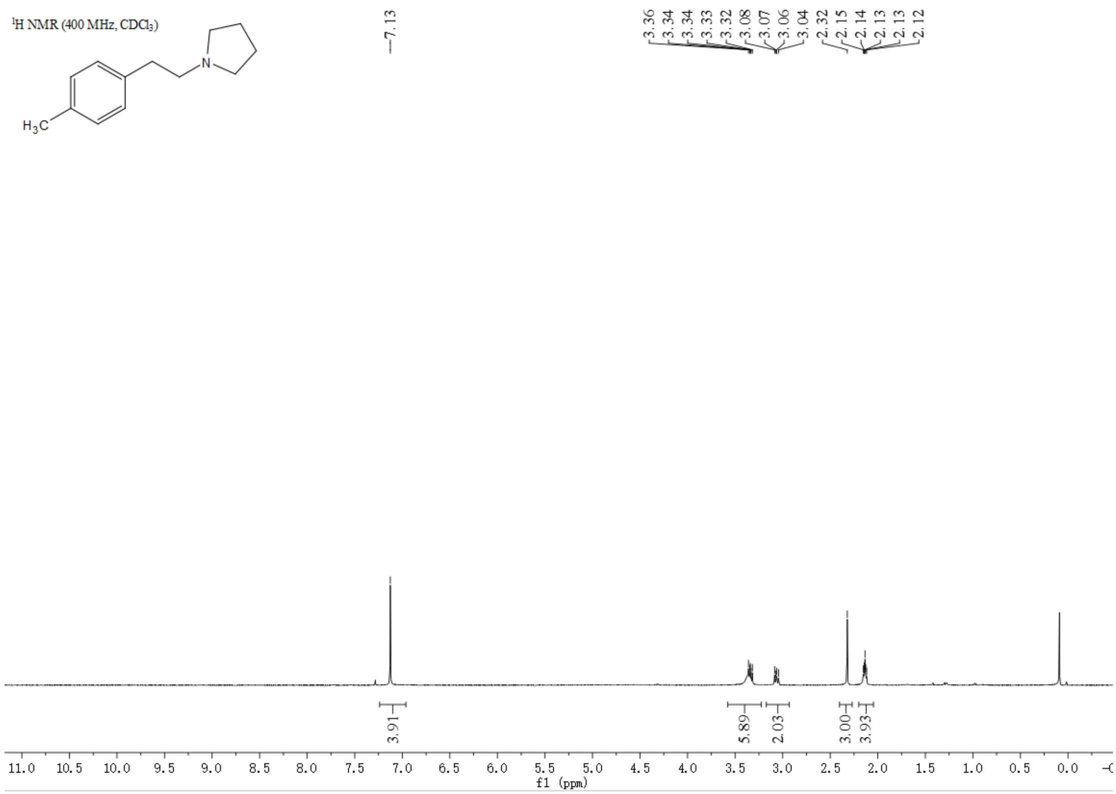

HRMS spectrum of 7o

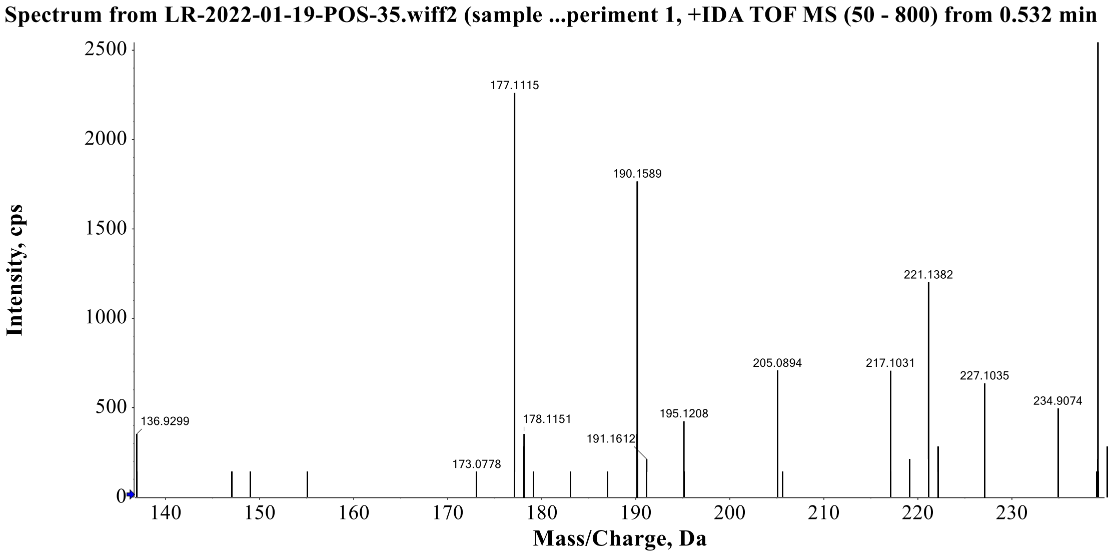

^{1}H NMR spectrum of 7p

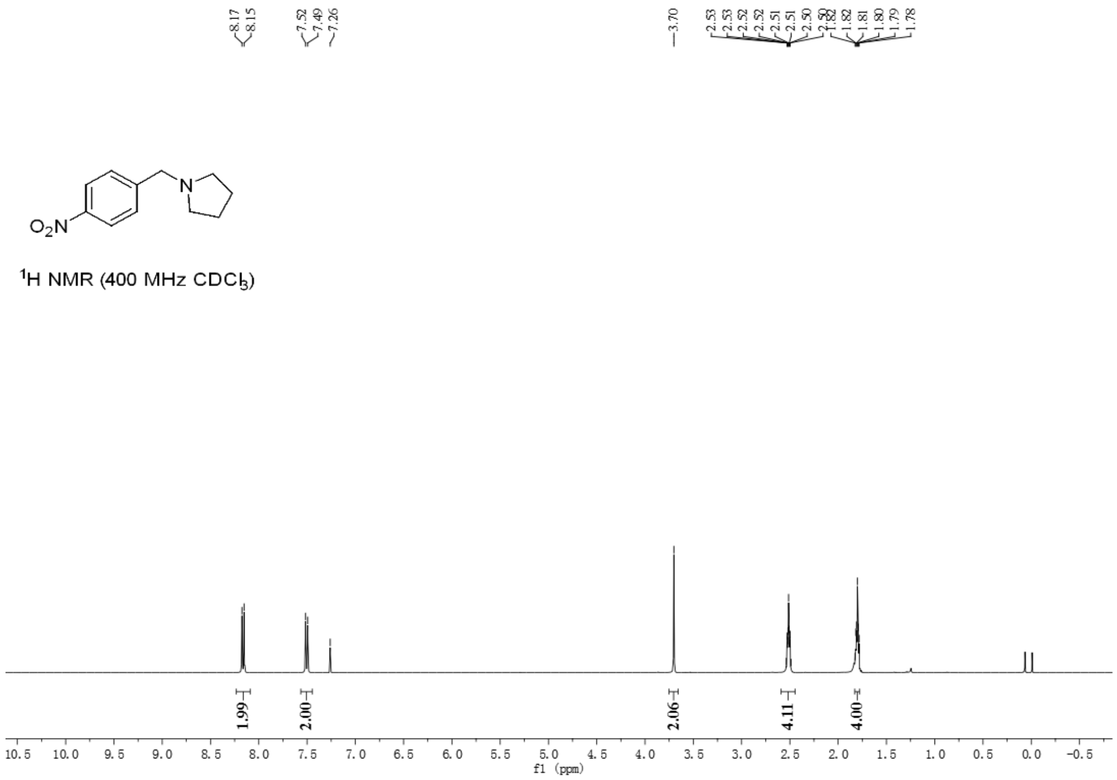

HRMS spectrum of 7p

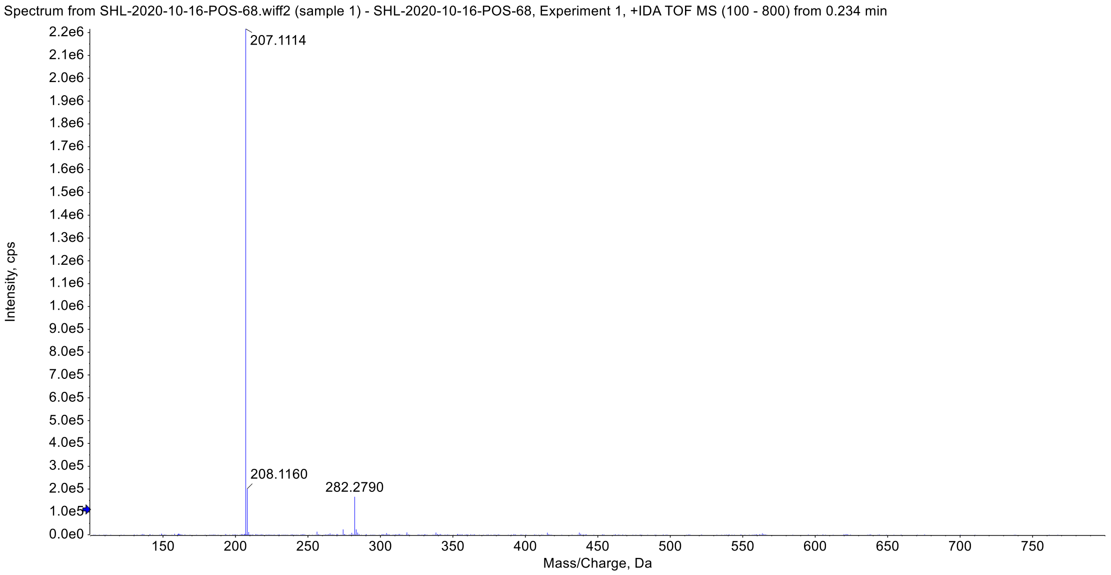

^{1}H NMR spectrum of 7q

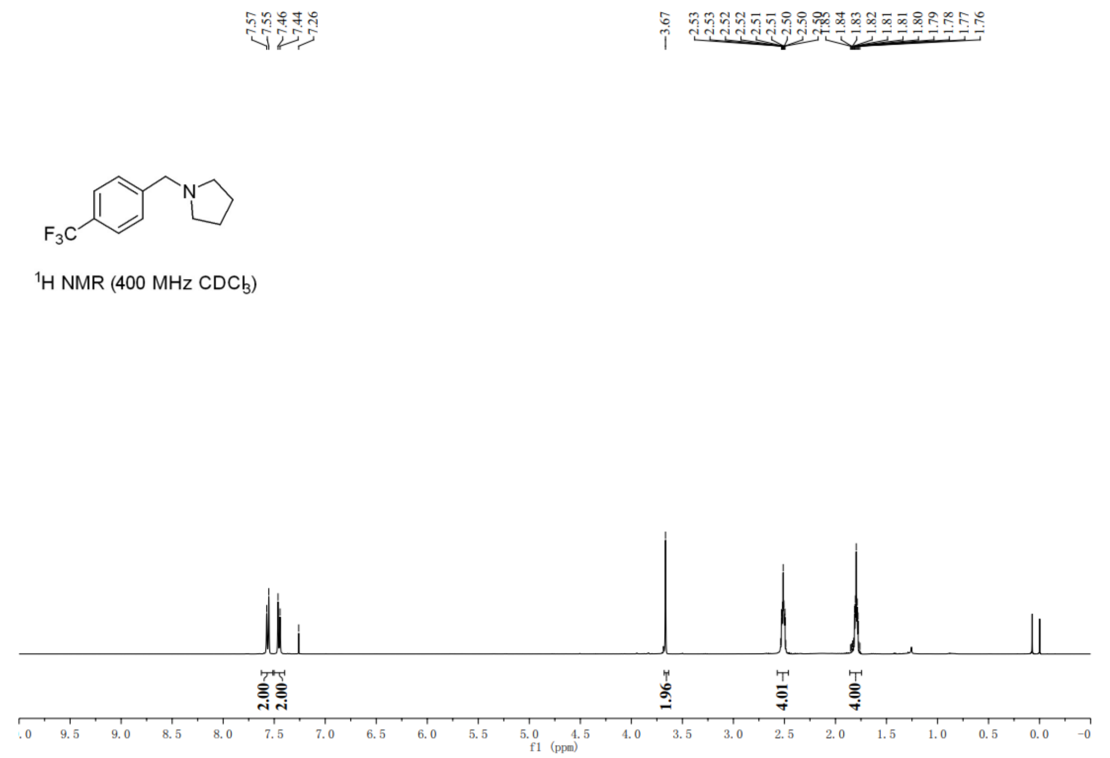

HRMS spectrum of 7q

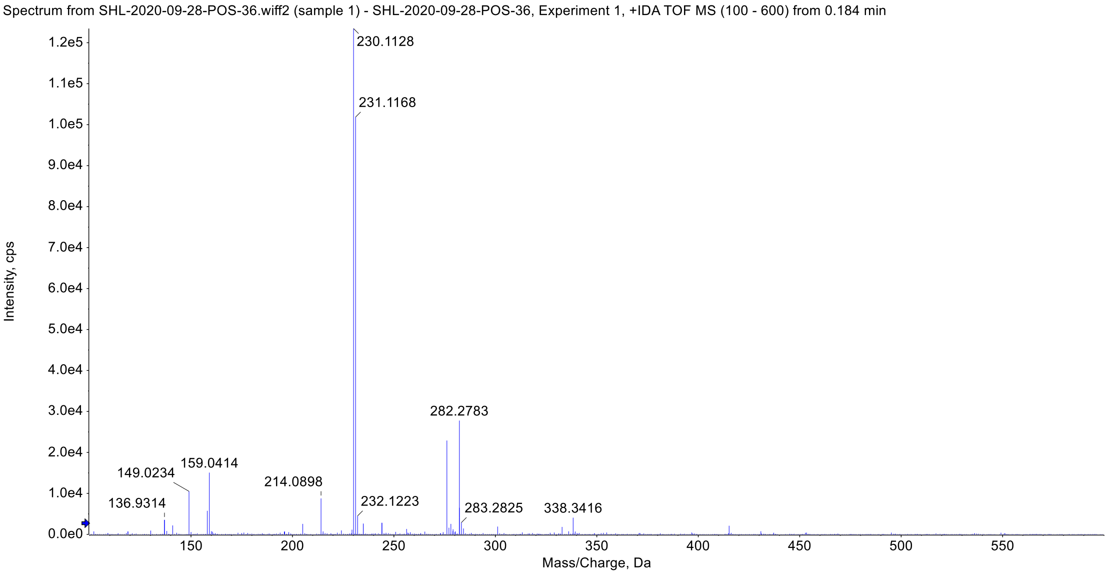

^{1}H NMR spectrum of 7r

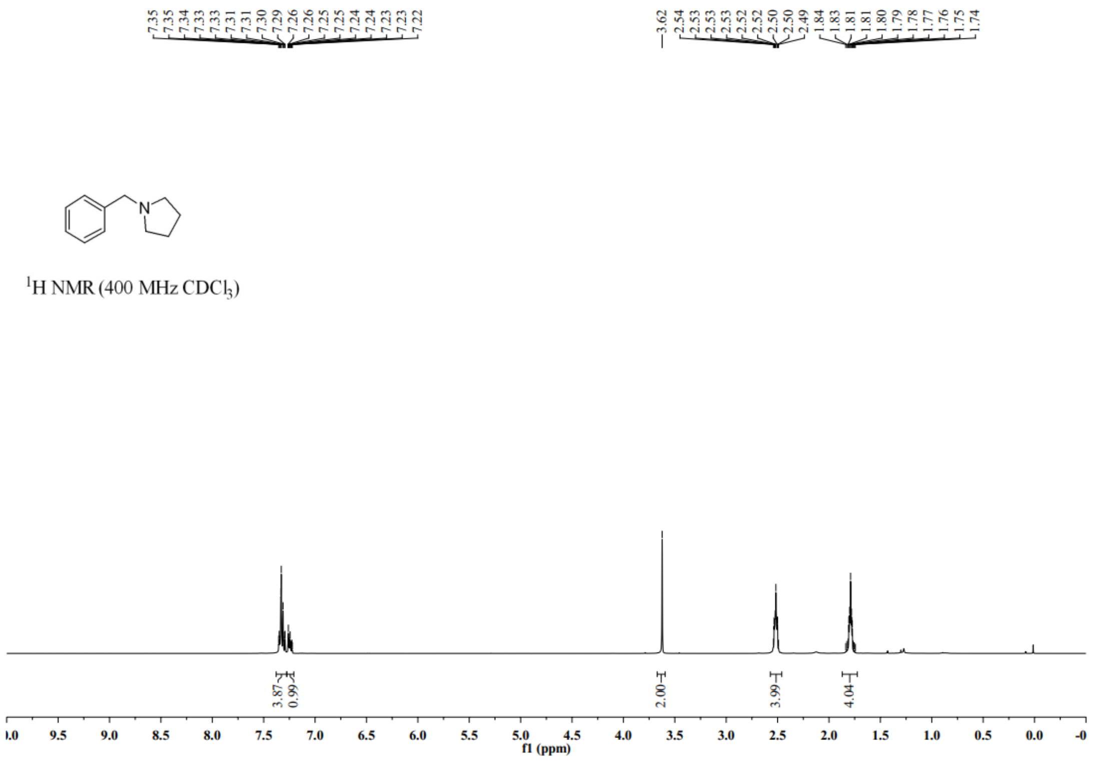

HRMS spectrum of 7r

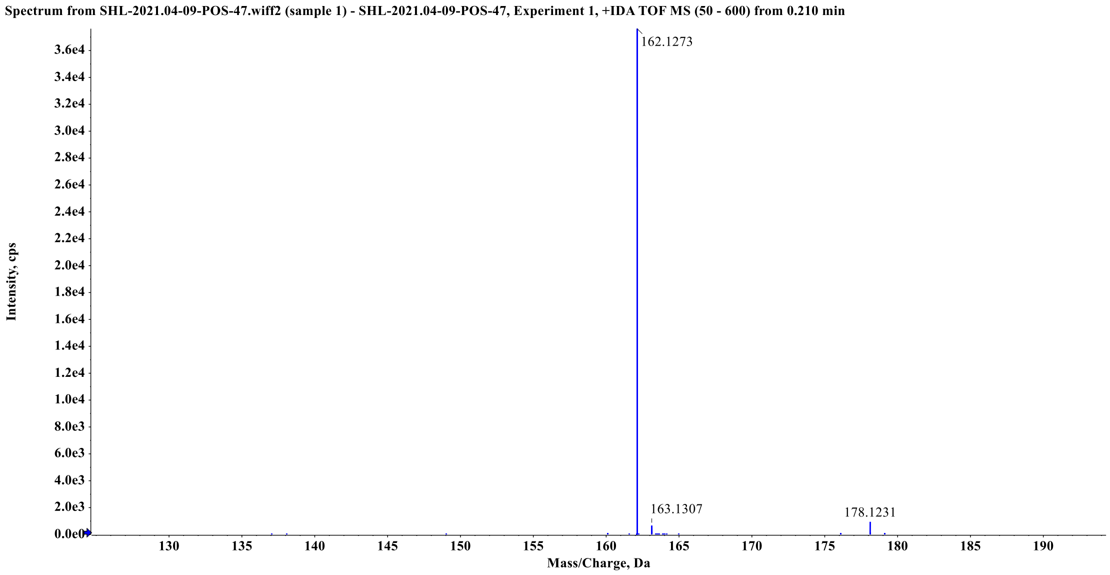

^{1}H NMR spectrum of 7s

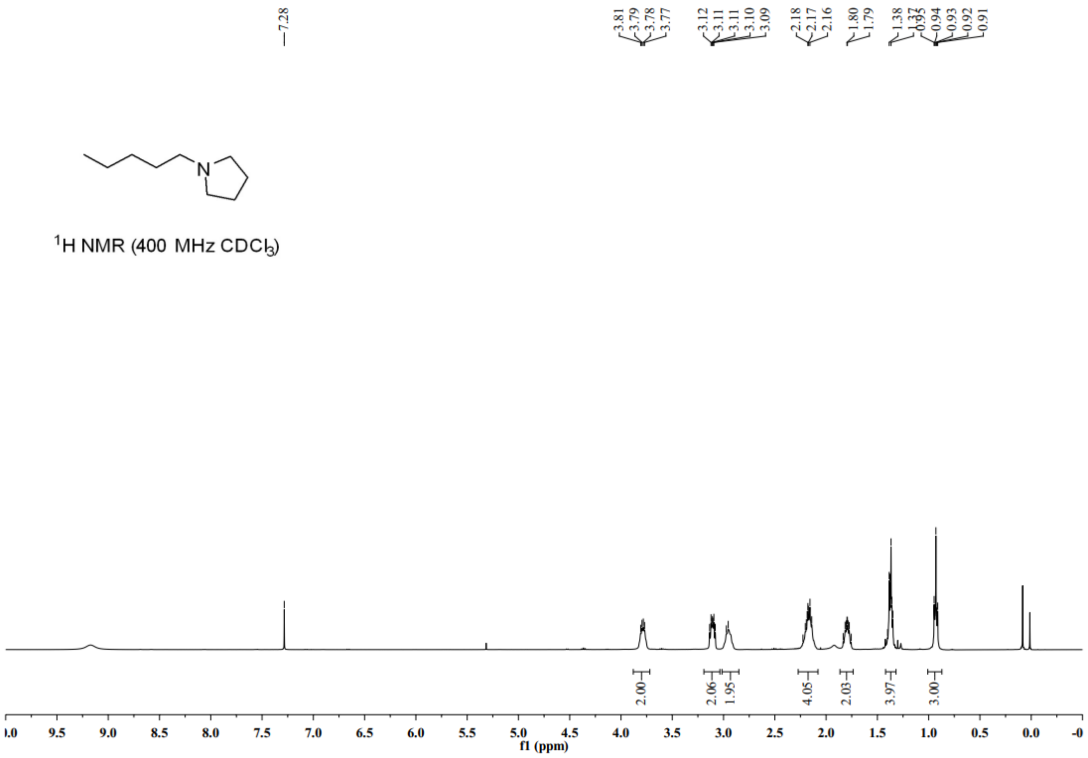

HRMS spectrum of 7s

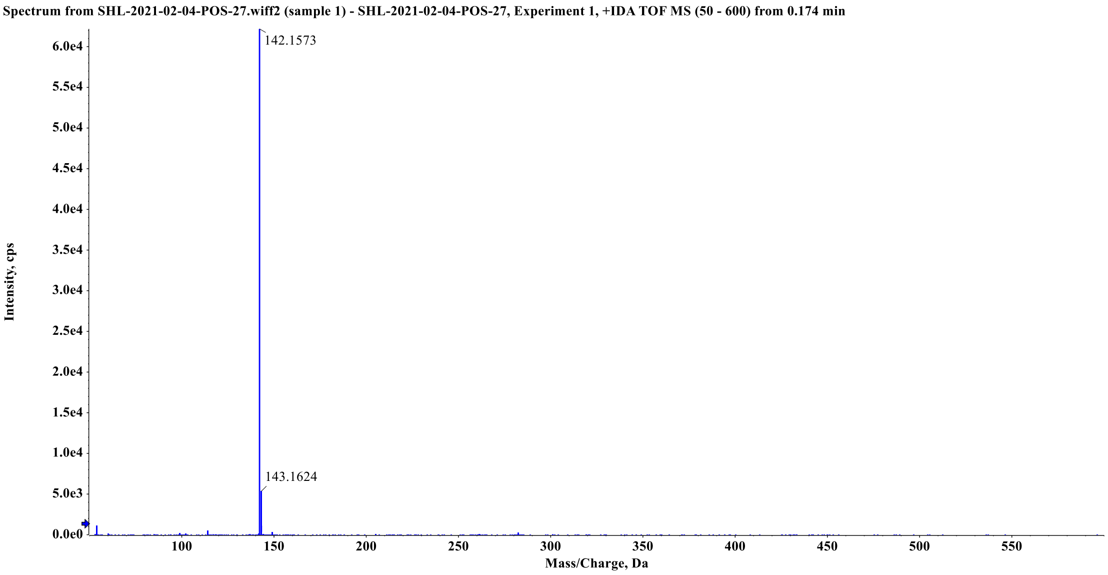

^{1}H NMR spectrum of 7t

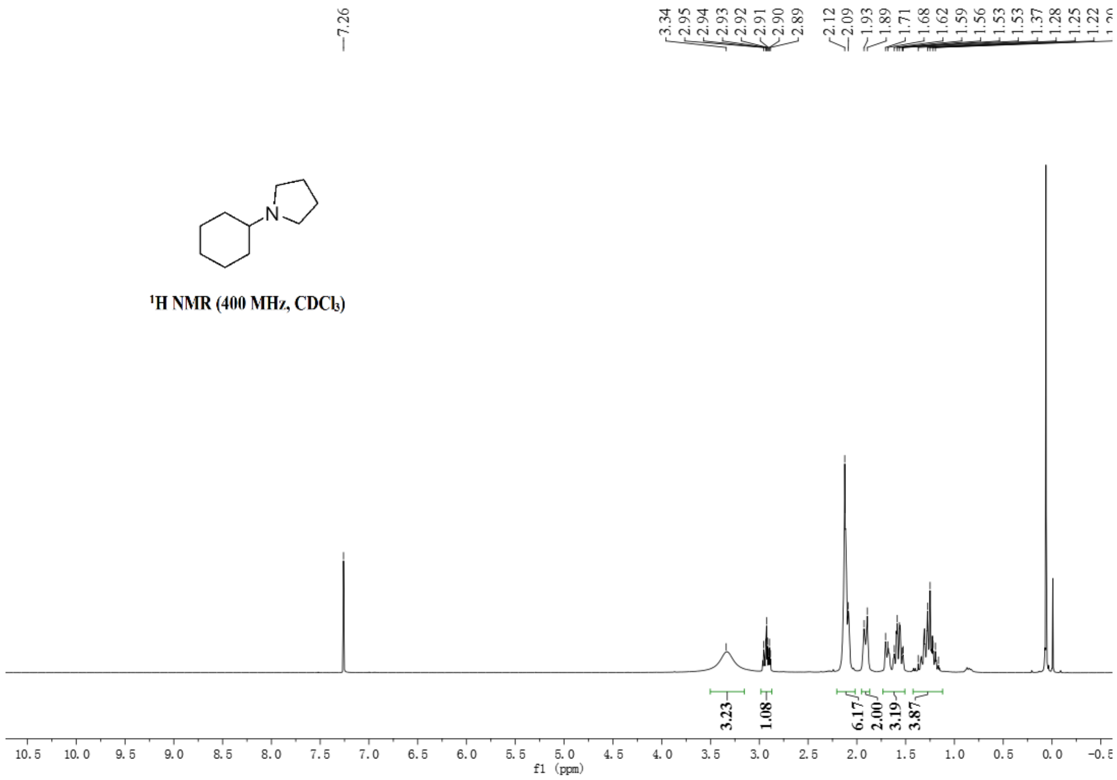

HRMS spectrum of 7t

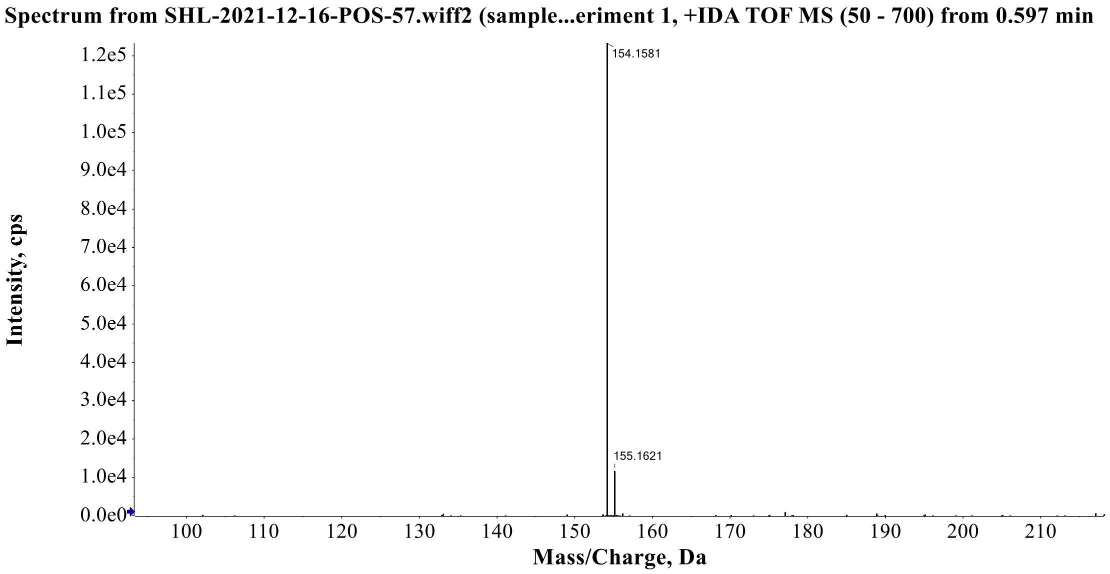

^{1}H NMR spectrum of 7u

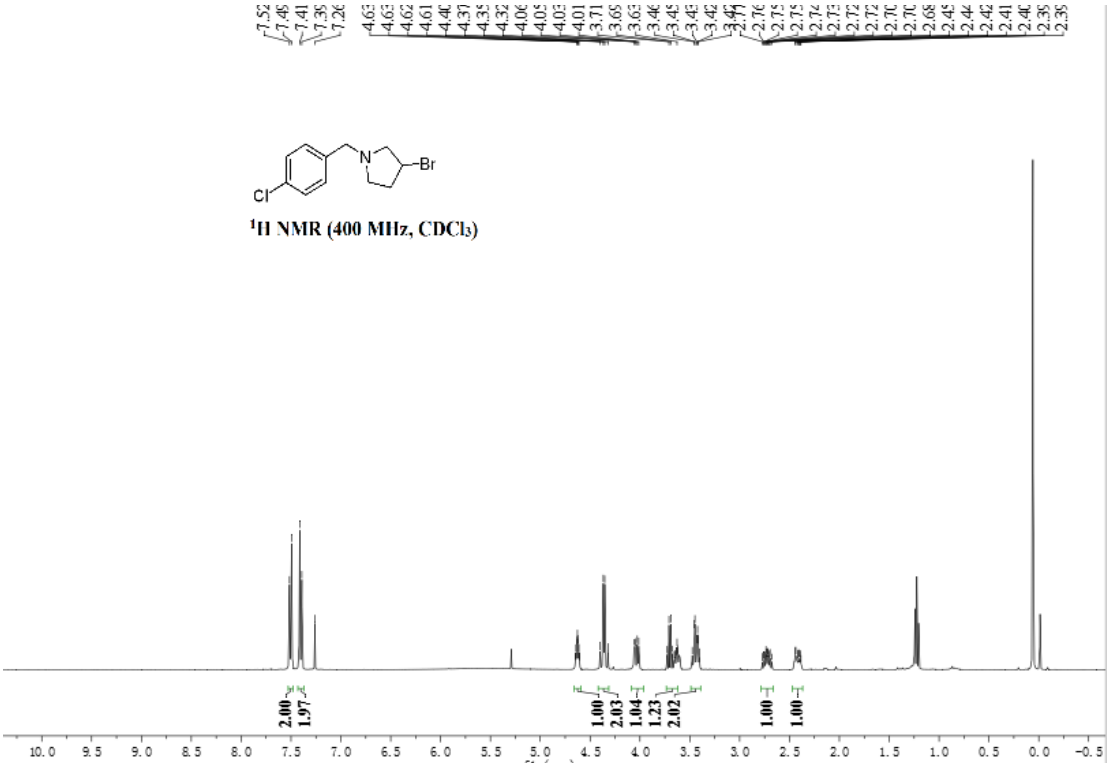

^{13}C NMR spectrum of 7u

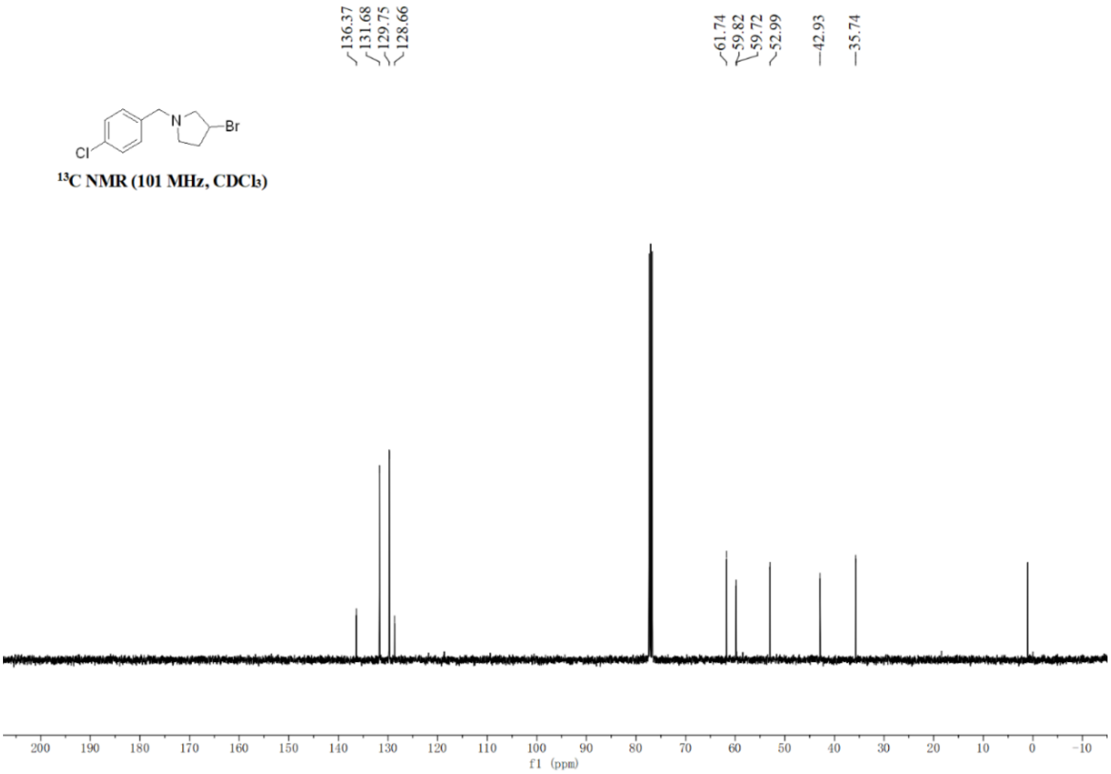

HRMS spectrum of 7u

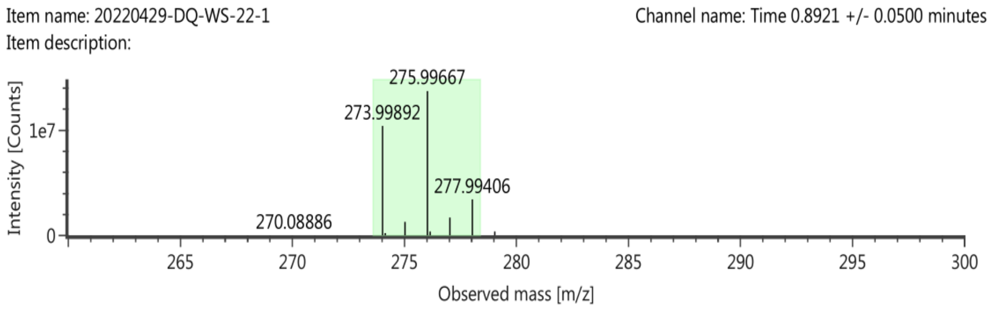

Supplement: Supplementary file 1 [file molecules-27-04698-s001.zip › molecules-1815042-supplementary.pdf]
